# Development of Candied Strawberry Products via Neural Network-Optimized Sugar-Infiltration Processes

**DOI:** 10.3390/foods15213871

**Published:** 2026-10-29

**Authors:** Qing Gao, Langsha Duan, Ai Xiao, Jiaxiu Wen, Nanxin Wang, Shuxin Ye, Jinsong He

**Affiliations:** College of Food Science and Technology, Yunnan Agricultural University, Kunming 650201, China

**Keywords:** candied strawberry products, high hydrostatic pressure, ultrasound, sugar infiltration, response surface methodology, back-propagation neural network

## Abstract

Conventional thermal processing of candied strawberries causes severe nutrient degradation and impairs the nutritional quality of the final products. To address this problem, high hydrostatic pressure (HHP) and ultrasound (US) were adopted as non-thermal auxiliary techniques for sugar-infiltration treatment in this study. Single-factor experiments were performed, and response surface methodology (RSM) and back-propagation neural networks (BPNN) were applied to optimize the processing parameters, with the sensory score used as the evaluation indicator. The optimization performance of the two models was then compared. Thermally processed candied strawberries served as the control group, and the moisture, total sugar, total acid, total phenolic, and total flavonoid contents of all sample groups were determined. The BPNN produced smaller relative errors than RSM in both HHP-assisted (0.95% vs. 1.15%) and US-assisted (0.47% vs. 0.68%) sugar-infiltration optimization, which confirms its superior predictive capability. The optimal HHP-assisted parameters were an infiltration time of 7.70 min, a treatment pressure of 258.00 MPa, and a sugar-solution concentration of 45.00%. The optimal US-assisted conditions were a treatment time of 50.00 min, an ultrasonic power of 80.00%, and a sugar-solution concentration of 45.00%. No significant differences in moisture content were observed among the three processing groups (thermal processing, 19.45 ± 0.35 g/100 g; US treatment, 19.51 ± 0.26 g/100 g; HHP treatment, 19.88 ± 0.21 g/100 g). The non-thermal treatments markedly enhanced nutrient retention: the total phenolic contents of the US- and HHP-treated samples reached 4.85 mg/g and 4.89 mg/g, respectively, which were 6.8-fold higher than that of the thermally processed samples (0.71 mg/g), and the corresponding total acid contents were 5.93 g/kg and 6.19 g/kg, approximately 9.5-fold higher than that of the thermal group (0.64 g/kg). In contrast, the thermally processed samples showed significantly higher total sugar contents than the non-thermal treatments. Both HHP and US processing effectively suppressed the thermal degradation of phenolic compounds and organic acids and reduced the total sugar content by approximately 24%. These results indicate that non-thermal processing is an effective route for manufacturing low-sugar, high-nutrition candied strawberry products.

## 1. Introduction

*Fragaria × ananassa* Duch. (strawberry) is a commercially popular fruit valued for its attractive color, desirable sweet–sour taste, and abundant nutrients such as vitamin C, saccharides, and minerals. It also exerts several health-related functions, including antioxidant activity, digestion promotion, and eyesight protection [1]. Nevertheless, fresh strawberries contain 90–95% moisture and are susceptible to mechanical damage and microbial spoilage, and they therefore deteriorate rapidly after harvest [2]. Concentrated harvest periods combined with a short shelf life frequently result in considerable economic losses [3]. Processing fresh strawberries into candied fruit products is an effective strategy for extending shelf life, reducing post-harvest losses, and diversifying consumer options.

Sugar infiltration is a core unit operation in candied-fruit production [4]. Conventional thermal sugar infiltration relies on high temperatures and long treatment times, which leads to substantial nutrient loss and inconsistent product quality [5]. Emerging non-thermal auxiliary technologies, such as high hydrostatic pressure (HHP) [6], vacuum treatment [7], and ultrasound (US) [8], have attracted increasing attention in food processing. Because of their mild processing conditions and low energy consumption, these technologies accelerate the penetration of the sugar solution into fruit tissues while preserving the original sensory and nutritional attributes of candied fruits; they therefore represent a promising route for manufacturing high-quality preserved fruit. However, the efficiency of non-thermal sugar infiltration is governed by the coupled effects of several processing parameters, including pressure, treatment time, and ultrasonic power; consequently, systematic optimization approaches are required to determine the optimal parameter combination.

Response surface methodology (RSM) based on a Box–Behnken design (BBD) relies on quadratic polynomial fitting; it is efficient for experimental design and adequately interprets factor interactions, but its fitting accuracy is limited for complex, highly nonlinear multi-factor problems. In contrast, back-propagation neural networks (BPNN) perform well in nonlinear approximation and prediction [9]. Previous comparative studies have demonstrated the respective strengths and limitations of RSM and artificial neural network (ANN)-type models in food-process modeling [10,11], although BPNN performance depends strongly on the quality of the training samples and on the parameter configuration. RSM is superior for analyzing interactions among variables, whereas BPNN achieves high-precision nonlinear prediction; the two methods therefore have complementary optimization merits and merit comparative assessment within a unified experimental system.

Orthogonal tests and response-surface approaches dominate existing process-optimization studies on candied strawberries [12,13], and machine-learning algorithms have also been applied to evaluate fruit quality attributes in related fruit-processing research [14]. Although previous food-processing studies have confirmed the higher prediction accuracy of BPNN relative to response-surface methods [11], most available RSM–BP comparisons address extraction processes that target bioactive-component yields rather than fruit-based osmotic-infusion processes. Moreover, few preserved-fruit studies have systematically compared the physicochemical and bioactive-substance differences between non-thermally optimized products and thermally processed control samples. In particular, the sugar infiltration of candied strawberries has mostly been studied under conventional thermal osmosis, and few studies have constructed BP neural network models to separately optimize the parameters of HHP-assisted and ultrasound-assisted sugar infiltration. Consequently, the quality variation of candied strawberries under these two non-thermal treatments has not been fully clarified, and targeted parameter-optimization strategies are lacking.

To address this gap, the novelty of this work lies in the separate modelling and parameter optimization of HHP-assisted and ultrasound-assisted sugar infiltration using BP neural networks, together with the systematic comparison of physicochemical quality between thermally processed and non-thermally processed candied strawberries. Unlike previous studies that mainly adopted RSM for process optimization of fruit osmotic dehydration, the present work uses a BP neural network to capture the nonlinear relationships between processing variables and quality indicators, thereby providing an alternative approach for parameter optimization of the non-thermal sugar infiltration of strawberry preserves. Accordingly, the objectives of this work were to establish BP neural network models for the HHP- and ultrasound-assisted sugar infiltration of candied strawberries, to determine the corresponding optimal processing parameters, and to compare the physicochemical quality of the resulting products with that of thermally processed samples. The results are expected to provide a theoretical basis and technical reference for the application of non-thermal processing technologies in the preserved-fruit industry.

## 2. Materials and Methods

### 2.1. Materials, Reagents, and Instruments

#### 2.1.1. Materials and Reagents

Fresh strawberries were purchased from Aishang Fruit Supermarket (Panlong District, Kunming, China), and food-grade white granulated sugar was obtained from Yunnan Nongjiyuan Biotechnology Co., Ltd., Kunming, China. Food-grade calcium chloride was supplied by Zhejiang Dacheng Calcium Industry Co., Ltd., Quzhou, China, and food-grade citric acid was obtained from Qixing Lemon Technology Co., Ltd., Linyi, China, Food-grade ascorbic acid was purchased from Guzhiwei Biotechnology Guangzhou, China, while food-grade glucose and high-fructose corn syrup were sourced from domestic food-grade suppliers. Analytical-grade hydrochloric acid and methylene blue were supplied by Tianjin Zhiyuan Chemical Reagent Co., Ltd., Tianjin, China, and analytical-grade methyl red, sodium hydroxide, potassium sodium tartrate, and potassium ferrocyanide were obtained from Tianjin Fengchuan Chemical Reagent Science and Technology Co., Ltd. Tianjin, China, Analytical-grade anhydrous ethanol, copper sulfate, and sodium carbonate were provided by Guangdong Guanghua Sci-Tech Co., Ltd. Shantou, China, Gallic acid and rutin reference standards were obtained from Shanghai Yuanye Bio-Technology Co., Ltd. Shanghai, China.

#### 2.1.2. Experimental Equipment

An ATY224 electronic analytical balance was manufactured by Shimadzu Corporation (Kyoto, Japan), and an SK3200HF ultrasonic cleaner was produced by Shanghai Kedao Ultrasonic Instrument Co., Ltd. (Shanghai, China). A 101 forced-air drying oven was supplied by Beijing Yongguangming Medical Instrument Factory (Beijing, China), and a V-5600 UV–Vis spectrophotometer was manufactured by Shanghai Yuanxi Instrument Co., Ltd. (Shanghai, China). A TGL-1650 high-speed centrifuge was obtained from Shuke Instrument Co., Ltd. (Chengdu, China), and an HH-8 constant-temperature water-bath apparatus was supplied by Jintan Chengxi Zhengrong Experimental Instrument Factory (Jintan, China). A household induction cooker was manufactured by Midea (Yunnan, China), and an HPP.L1-600/5 high hydrostatic-pressure system was supplied by Tianjin Huatai Senmiao Biotechnology Co., Ltd. (Tianjin, China). An FS-300 manual plastic sealer was manufactured by Wenzhou Zhengxiong Machinery Co., Ltd. (Wenzhou, China). The soluble solids content was determined with a JK-119 handheld refractometer (Chengdu Haochuang Optoelectronic Instrument Co., Ltd., Chengdu, China).

### 2.2. Experimental Methods

#### 2.2.1. Preparation Process of Candied Strawberry Products

**Figure 1:**
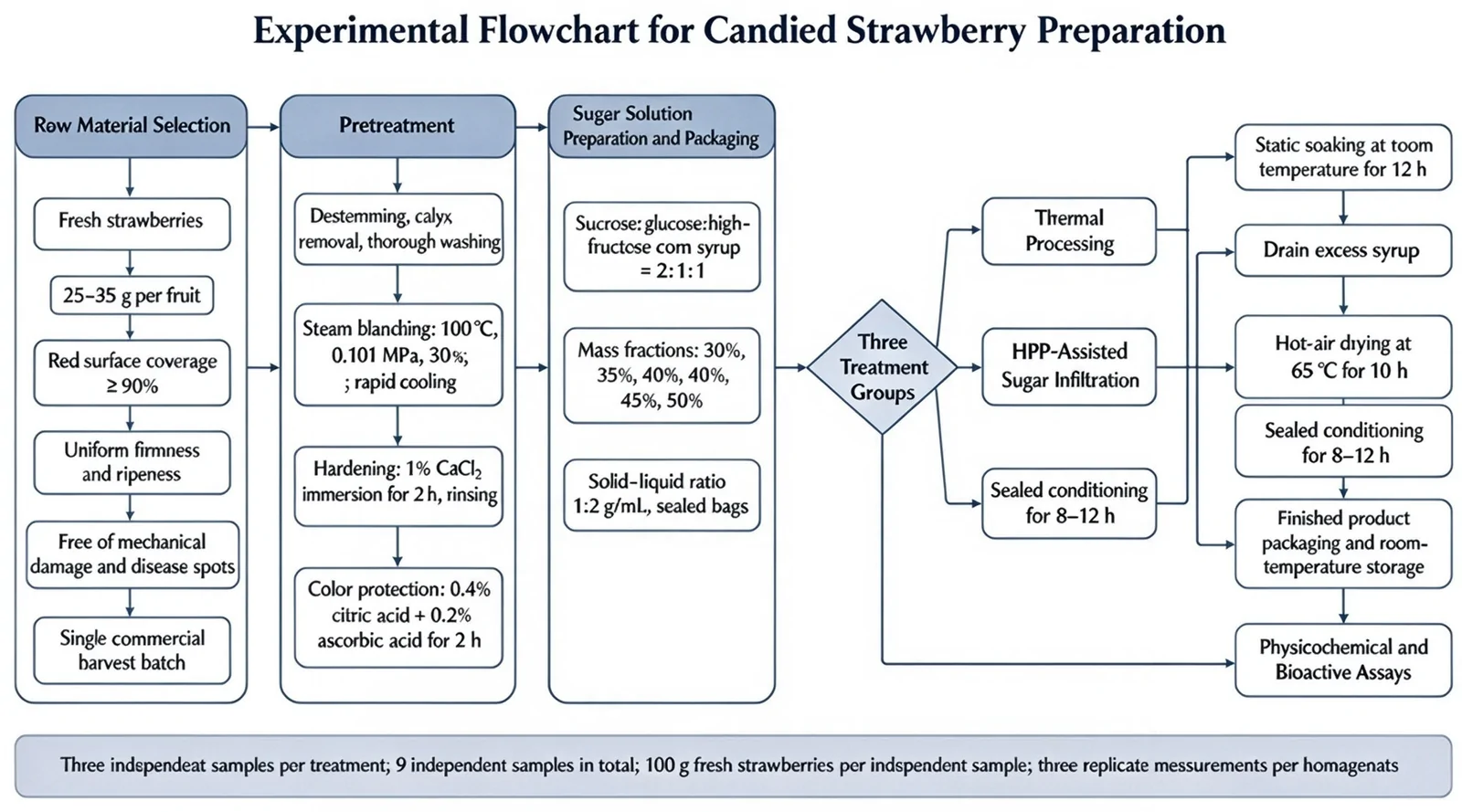
Experimental flowchart for candied strawberry preparation.

Strawberries at the full-ripening stage were selected on the basis of visual appearance and soluble solids content (SSC). Fruits with uniformly full-red skin and no mechanical injury or rot were first screened visually, and their SSC was measured with a handheld refractometer. Only fruits with an SSC of 8.0–10.0 °Brix were collected for candied-fruit preparation. A total of approximately 27–36 fresh strawberries (900 g) were used for candied-fruit preparation in this study. Of these, 9–12 fruits (300 g) were allocated to the thermal-processing group, 9–12 fruits (300 g) to the HHP-assisted sugar-infiltration group, and 9–12 fruits (300 g) to the US-assisted sugar-infiltration group. Each treatment comprised three independent biological replicates, and each biological replicate consisted of 3–4 fresh strawberries (100 g). The fruits were destemmed, the calyxes were removed, and the samples were washed thoroughly. Steam blanching was performed at 93 °C under atmospheric pressure for 30 s, after which the samples were rapidly cooled to ambient temperature in cold water. Hardening was carried out by immersion in 1% CaCl_2_ solution for 2 h, followed by rinsing to remove residual reagent, and color protection was achieved by immersion in a mixed solution of 0.4% citric acid and 0.2% ascorbic acid for 2 h. A compound sugar solution was then prepared at a mass ratio of sucrose–glucose–high-fructose corn syrup = 2:1:1, and the solution concentration was adjusted to 30%, 35%, 40%, 45%, or 50% (mass fraction). Strawberries and sugar solution were loaded into sealed bags at a solid–liquid ratio of 1:2 (g/mL), and three treatment groups were prepared: thermal processing, HHP-assisted sugar infiltration, and US-assisted sugar infiltration. After sugar infiltration, the samples were soaked at room temperature for 12 h, and the excess syrup was drained off. Drying was performed in a forced-air drying oven at 65 °C for 10 h.; the oven used circulating ambient air without humidity control, and the relative humidity inside the chamber varied dynamically during drying. The dried candied-fruit samples were sealed and tempered for 8–12 h to achieve a uniform moisture distribution, after which the finished products were packed and stored at room temperature for the subsequent analyses. The sample preparation procedure is illustrated in Figure 1.

#### 2.2.2. Thermally-Processed Control Group

Water-bath heating was used to simulate conventional thermal sugar infiltration of candied strawberries. The sugar solution was prepared at the concentrations optimized in the preliminary tests, and strawberries and sugar solution were mixed at a solid–liquid ratio of 1:2 (g/mL) and incubated in a 65 °C water bath for 5 h. The subsequent draining, drying, and packaging operations were identical to those applied to the non-thermal groups.

#### 2.2.3. Single-Factor Experiments

(1) Single-Factor Experiment for HHP-Assisted Sugar Infiltration

Single-factor trials were conducted for the HHP-assisted process. The infiltration time was varied over 2, 4, 6, 8, and 10 min, the treatment pressure over 100, 150, 200, 250, and 300 MPa, and the sugar-solution concentration over 40%, 45%, and 50%, with the sensory score used as the response variable. Suitable factor-level ranges were then identified for the subsequent BBD experiments.

(2) Single-Factor Experiment for US-Assisted Sugar Infiltration

Single-factor trials were likewise conducted for the US-assisted process. The ultrasonic treatment time was varied over 30, 40, 50, 60, and 70 min, the ultrasonic power over 70%, 80%, and 90%, and the sugar-solution concentration over 30%, 35%, 40%, 45%, and 50%, with the sensory score used as the response value. Suitable parameter ranges were then determined for the subsequent response-surface optimization.

#### 2.2.4. Response-Surface Methodology Experiments

(1) HHP-Assisted Sugar-Infiltration Response-Surface Experiment

A three-factor, three-level BBD was adopted. sugar infiltration time (X_1_), treatment pressure (X_2_), and sugar-solution concentration (X_3_) were selected as the independent variables, and the sensory score (Y_1_) was set as the response value. The factor levels are summarized in Table 1. The center-point experiment was performed in quintuplicate to estimate the pure experimental error.

(2) US-Assisted Sugar-Infiltration Response-Surface Experiment

A three-factor, three-level BBD was performed. Ultrasonic treatment time (X_4_), ultrasonic power (X_5_), and sugar-solution concentration (X_6_) were treated as the independent variables, and the sensory score (Y_2_) was the response variable. The factor levels are listed in Table 2. The center-point experiment was performed in quintuplicate to estimate the pure experimental error.

#### 2.2.5. Sensory Evaluation

Ten panelists (aged 21–22 years; three males and seven females) were recruited from undergraduate students for the sensory evaluation. Panelist screening covered health status, the absence of taste and smell disorders, the ability to distinguish differences in basic taste and texture, availability for all training and test sessions, and willingness to comply with the test requirements. Before the formal evaluation, the panelists completed a 4-week training program consisting of two 1.5 h sessions per week. During training, they were instructed in the principles of sensory evaluation, the definition of each attribute, the scoring criteria, and the test procedures. Four attributes were evaluated: appearance, aroma, sweet–sour taste, and texture. The panelists practiced scoring with standard reference samples representing different quality grades and were required to align their scoring with the consensus criteria. Their competency and consistency were verified by scoring eight sets of duplicate reference samples; panelists who achieved a correlation coefficient of ≥0.80 for the duplicate sample scores and a total score deviation of less than 8 points were considered qualified and were selected for the formal sensory test. A unified 100-point scoring system was used, and the detailed criteria are presented in Table 3. All panelists signed an informed consent form before participating.

For each test session, candied strawberry samples were prepared under the same conditions and presented at room temperature. Each portion was approximately 10 g and was placed in an opaque disposable plastic cup. All samples were labeled only with random three-digit blind codes, and no information on treatment, identity, or order was provided to the panelists. The presentation order was fully randomized for each panelist to reduce order and carryover effects, and each panelist evaluated every sample twice with an appropriate interval between evaluations. Sensory evaluations were performed in individual sensory booths under strictly controlled conditions: the evaluation area was maintained at 22 ± 2 °C with uniform neutral white lighting, low noise, and good ventilation. The panelists rinsed their mouths thoroughly with purified drinking water and waited at least 60 s before evaluating the next sample to minimize residual taste interference, and they were instructed not to discuss the samples during evaluation. The final sensory score of each sample was the average score of all qualified panelists after extreme values had been excluded. The detailed scoring standards are provided in Table 3.

#### 2.2.6. Construction of BPNN Prediction Model and Parameter Optimization

(1) BPNN for HHP-Assisted Sugar Infiltration

**Figure 2:**
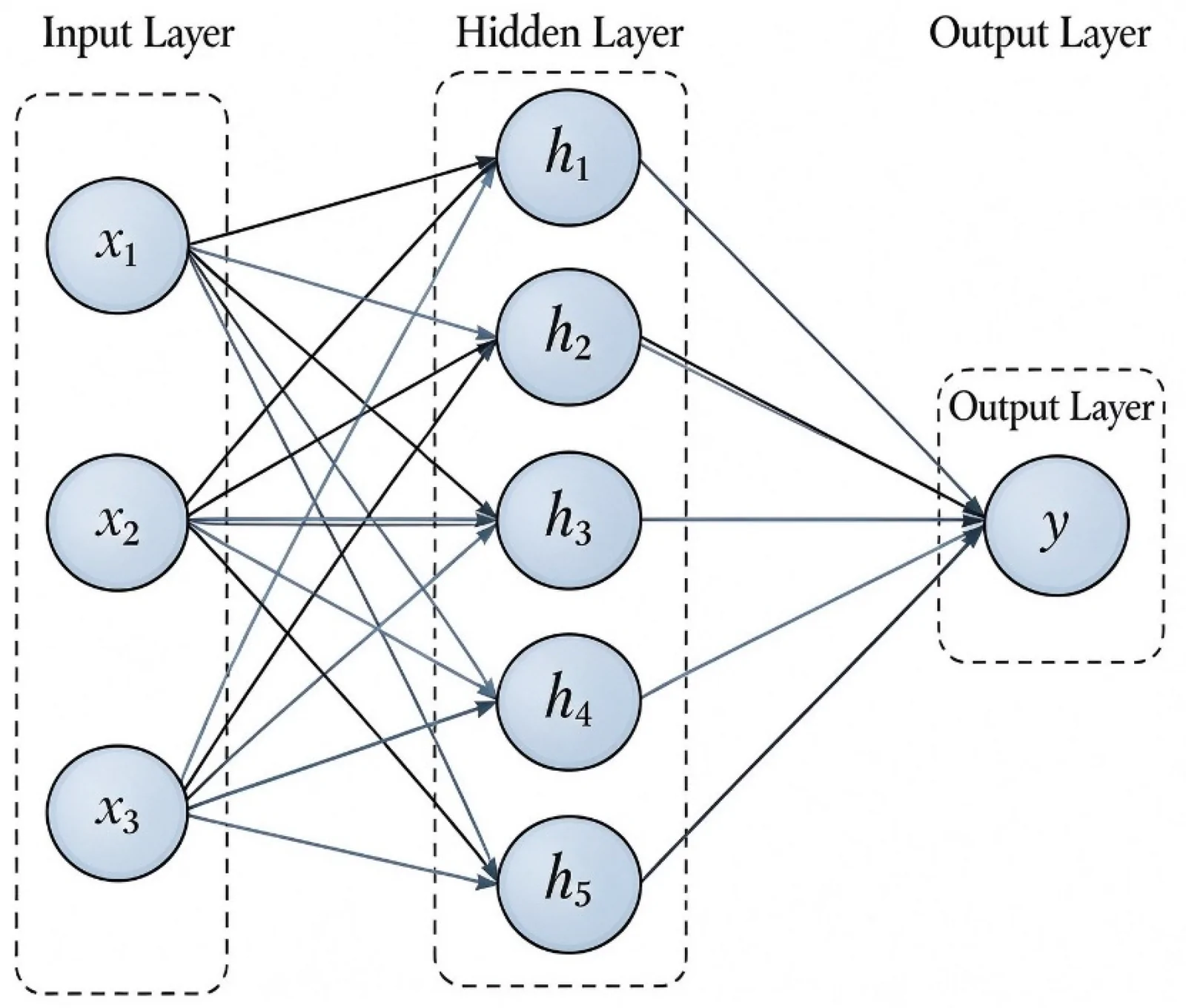
Schematic architecture of the BPNN model for ultrahigh-pressure osmotic dehydration.

The 17 groups of RSM experimental data were used for neural network training. Infiltration time, treatment pressure, and sugar-solution concentration were set as the input-layer neurons, and the sensory score was assigned to the single output neuron. MATLAB (R2024b) was used for model construction. The number of hidden-layer neurons was the key optimized hyperparameter, with a search range of 3–12 via the trial-and-error method [15]. Finally, five neurons in the single hidden layer achieved the best model performance. The topology diagram of this optimized 3-5-1 BPNN model for HHP-assisted sugar infiltration is shown in Figure 2. The learning rate ranged from 0.001 to 0.1 and the maximum number of training epochs from 10 to 100; the Levenberg–Marquardt (L-M) algorithm was used as the training function, tansig as the hidden-layer activation function, and purelin as the output-layer activation function. The dataset was randomly partitioned into 70% for training, 15% for validation, and 15% for testing, which yielded 12 training, 3 validation, and 2 test samples. Model performance was assessed using the mean square error (MSE) and the coefficient of determination (R^2^), and iterative training with cross-validation was performed to optimize the network hyperparameters. Grid-search global optimization was then carried out on the well-trained network, and the optimization results were compared with those obtained from RSM.

(2) BPNN for US-Assisted Sugar Infiltration

**Figure 3:**
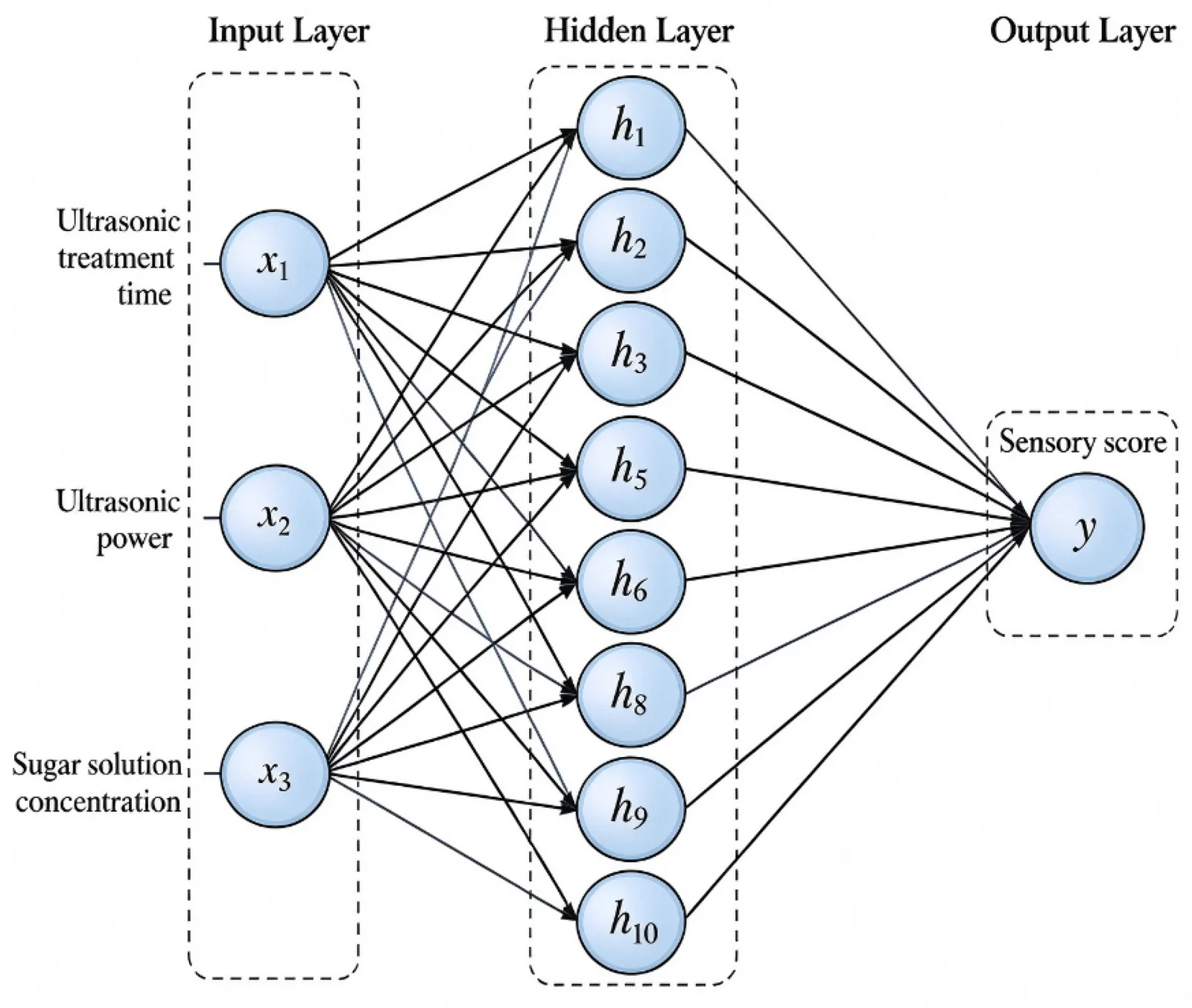
Schematic architecture of the BPNN model for ultra-high-pressure osmotic dehydration.

Identical modeling procedures described in Section 2.2.6 (1) were adopted. The BPNN for US-assisted sugar infiltration also used the 17 groups of BBD experimental data, and the whole dataset was randomly split in the same 70%:15%:15% ratio, which yielded 12 training, 3 validation, and 2 test samples. The same hyperparameter search ranges, training function, and activation-function configuration were adopted. Finally, ten neurons in a single hidden layer produced the optimal model performance. The topology of this optimized 3-10-1 BPNN model for US-assisted sugar infiltration is shown in Figure 3. Ultrasonic treatment time, ultrasonic power, and sugar-solution concentration served as the input variables, and the sensory score was used as the output variable for network training and parameter optimization.

#### 2.2.7. Determination of Physicochemical Indicators

(1) Moisture Content Determination

The moisture content was measured by oven drying with minor modifications according to Karim et al. [16]. Approximately 10.000 g of homogenized candied-fruit sample was weighed into a crucible and dried at 105 °C for 1 h; the samples were then cooled in a desiccator for 0.5 h and weighed. The drying–cooling–weighing cycle was repeated until a constant mass was reached (mass difference below 2 mg). All measurements were performed in triplicate.

(2) Total Sugar Determination

The total sugar content was determined with slight modifications following Kaur et al. [17]. A 10.000 g portion of homogenized candied-strawberry sample was soaked in distilled water for 1–2 h and homogenized, and the suspension was transferred to a 250 mL volumetric flask. A 10.00 mL aliquot of the sample solution was mixed with 30 mL distilled water and 5 mL hydrochloric acid and hydrolyzed in a 68–70 °C water bath for 10 min. After cooling, the solution was transferred, neutralized with sodium hydroxide solution using methyl red as the indicator, and diluted to volume, after which Fehling’s reagent titration was performed. Three technical replicates were implemented for each sample.

(3) Total Acid Determination

The total acid content was assayed according to Sharabiani et al. [18]. A 25.00 g portion of homogenized sample was transferred into a volumetric flask with distilled-water rinsing, heated in a boiling-water bath for 30 min, cooled, diluted to volume, and filtered. The filtrate was titrated with 0.10 mol/L NaOH solution. All determinations were carried out in triplicate.

(4) Total Phenolic Determination

The total phenolic content was quantified by the Folin–Ciocalteu colorimetric method modified from Shelke et al. [19]. Gallic acid standard solutions were prepared to construct the standard curve. Sample extracts were obtained by ultrasonic extraction with 70% ethanol (100 W, 30 min) followed by centrifugation at 6000 r/min for 20 min; the supernatants were collected, diluted, and subjected to Folin–Ciocalteu color development, and the absorbance was read at 765 nm. Each sample was measured in triplicate.

(5) Total Flavonoid Determination

The total flavonoid content was measured by the NaNO_2_–Al(NO_3_)_3_–NaOH colorimetric assay modified from Jia et al. [20]. Rutin standard solutions were prepared for calibration. The sample extracts were mixed with the color-reagent series, and the absorbance was recorded at 510 nm after full color development. All assays were repeated in triplicate.

#### 2.2.8. Data Statistical Analysis

All experiments were conducted in triplicate, and results were expressed as mean ± standard deviation. One-way analysis of variance (ANOVA) was performed to evaluate differences in the physicochemical indicators (moisture, total sugar, total acid, total phenolic, and total flavonoid contents) among the different processing treatments, and Tukey’s HSD post hoc test was used for pairwise multiple comparisons. The significance level was set at α = 0.05, and differences with *p* < 0.05 were regarded as statistically significant (*n* = 3). Microsoft Excel 2021 was used for basic data processing, Origin 2024 for graphical plotting, Design-Expert for the response-surface regression and ANOVA, and MATLAB R2024b for BP neural network training, calculation, and visualization.

## 3. Results

### 3.1. Single-Factor and Response-Surface Results for HHP- and US-Assisted Sugar Infiltration

#### 3.1.1. Single-Factor and Response-Surface Results for HHP-Assisted Sugar Infiltration

**Figure 4:**
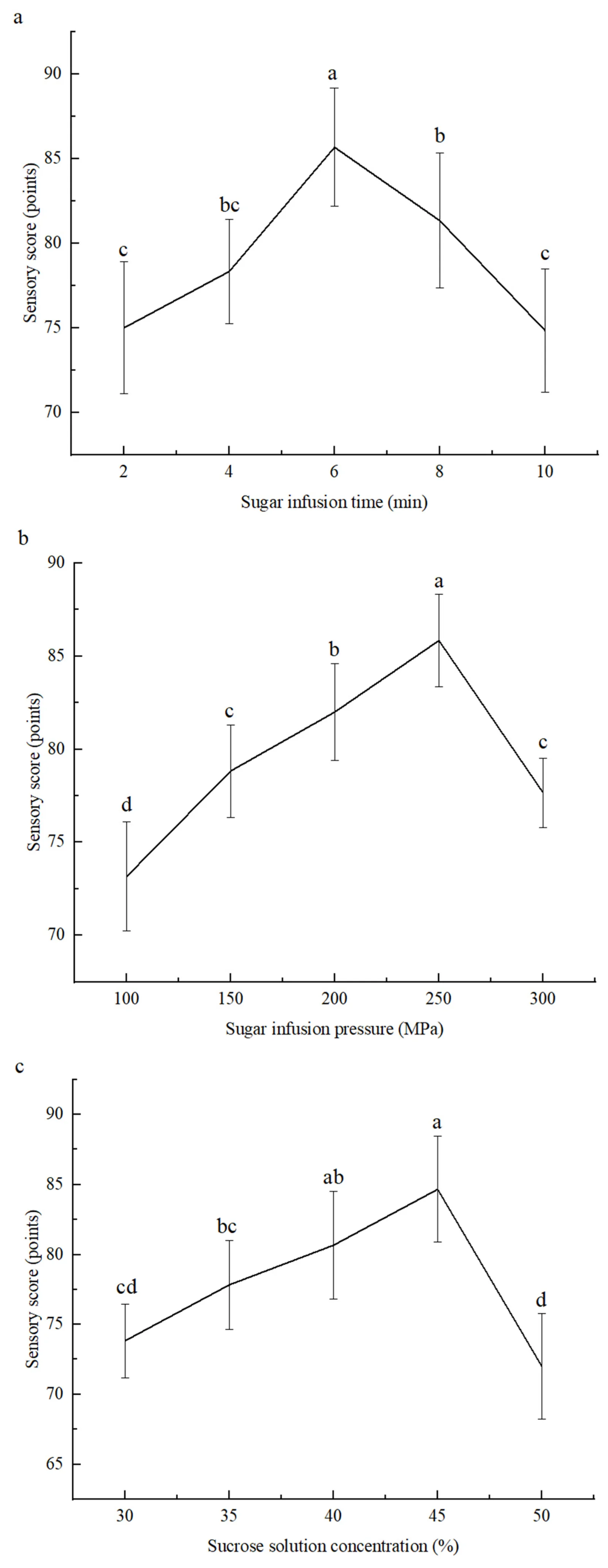
Main effect plots of single factors on sensory score of strawberry fruit during high-pressure assisted sugar infusion. (**a**) Effect of sugar infusion time on sensory score; (**b**) effect of sugar infusion pressure on sensory score; (**c**) effect of sucrose solution concentration on sensory score. Error bars represent standard deviation (*n* = 3). Note: Different lowercase letters indicate significant differences between treatments (*p* < 0.05).

The single-factor results are shown in Figure 4. The sensory score first increased and then decreased as the infiltration time, pressure, or sugar-solution concentration increased, and the maximum sensory score was obtained at an infiltration time of 6 min, a pressure of 250 MPa, and a sugar-solution concentration of 45%. The factor-level ranges for the BBD experiments were therefore set as follows: infiltration time 4, 6, and 8 min; pressure 200, 250, and 300 MPa; and sugar-solution concentration 40%, 45%, and 50%.

The quadratic regression model for HHP-assisted infiltration is given in Equation (1):

(1) $$Y_{1}=-89.06+4.74X_{1}+0.22X_{2}+5.91X_{3}-2.40\times 10^{-3}X_{1}X_{2}-5.00\times 10^{-3}X_{1}X_{3}\;\;\;+2.00\times 10^{-3}X_{2}X_{3}-0.32{X_{1}}^{2}-6.00\times 10^{-4}{X_{2}}^{2}-0.07{X_{3}}^{2}$$

where X_1_: infiltration time (min); X_2_: treatment pressure (MPa); X_3_: sugar-solution concentration (%); Y_1_: sensory score (points).

**Figure 5:**
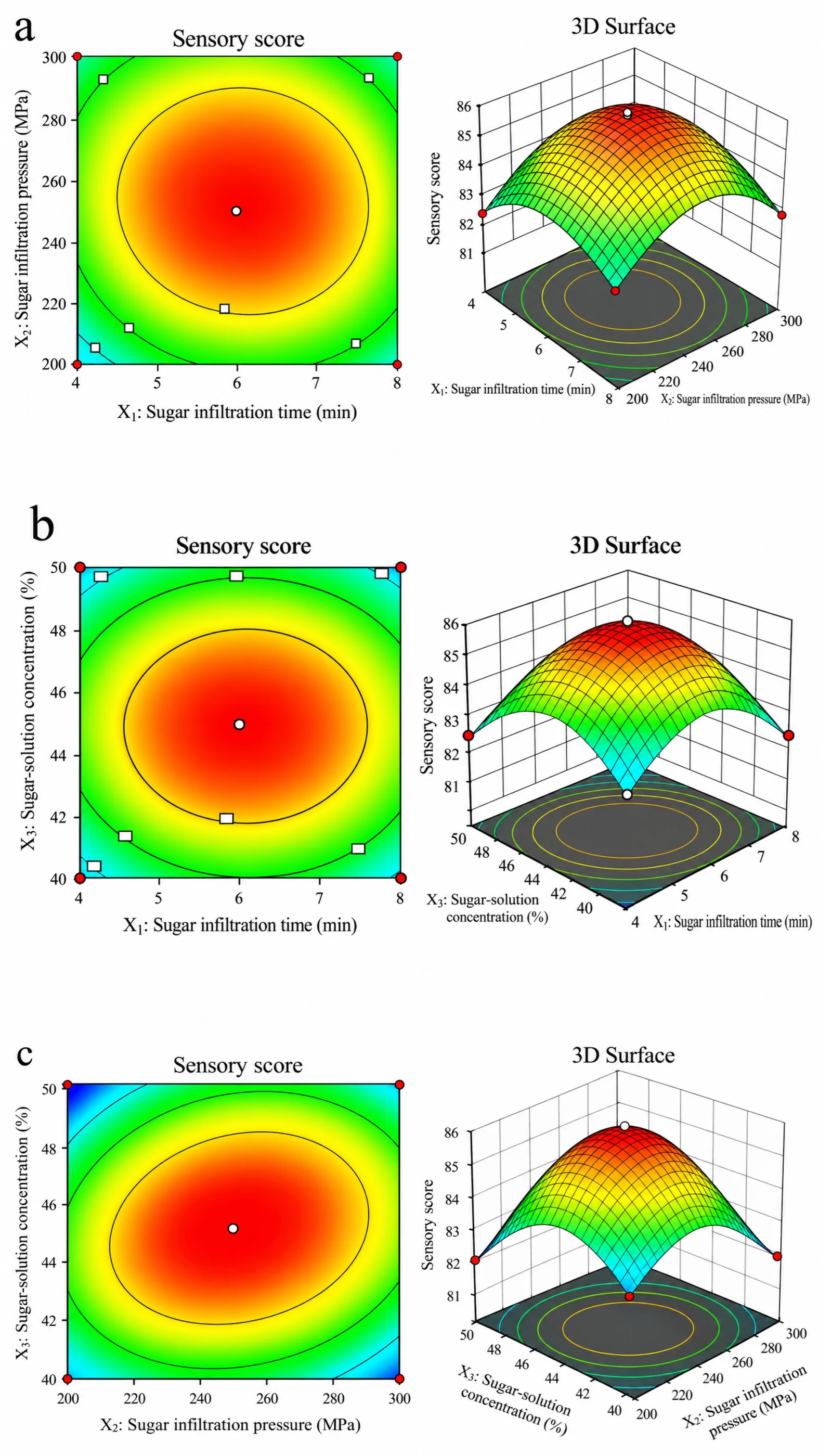
Contour plots and 3D response surface plots for sensory score of strawberry candied fruit. (**a**) Interaction between sugar infusion time and sugar infusion pressure; (**b**) interaction between sugar infusion time and sucrose solution concentration; (**c**) interaction between sugar infusion pressure and sucrose solution concentration.

ANOVA showed that the regression model was highly significant (*p* < 0.01, F = 105.30), with R^2^ = 0.99, and the quadratic terms were also highly significant (*p* < 0.01). The response-surface contour and 3D plots are displayed in Figure 5, and each factor produced an increase-then-decrease trend in the sensory score. The theoretical optimal HHP parameters calculated from the model were an infiltration time of 6.00 min, a pressure of 250.00 MPa, and a sugar-solution concentration of 45.00%, with a predicted sensory score of 85.82. Three parallel verification experiments gave measured sensory scores of 84.31, 84.86, and 85.32, with a mean of 84.83 ± 0.51 and a relative error of 1.15%.

#### 3.1.2. Single-Factor and Response-Surface Results for US-Assisted Sugar Infiltration

**Figure 6:**
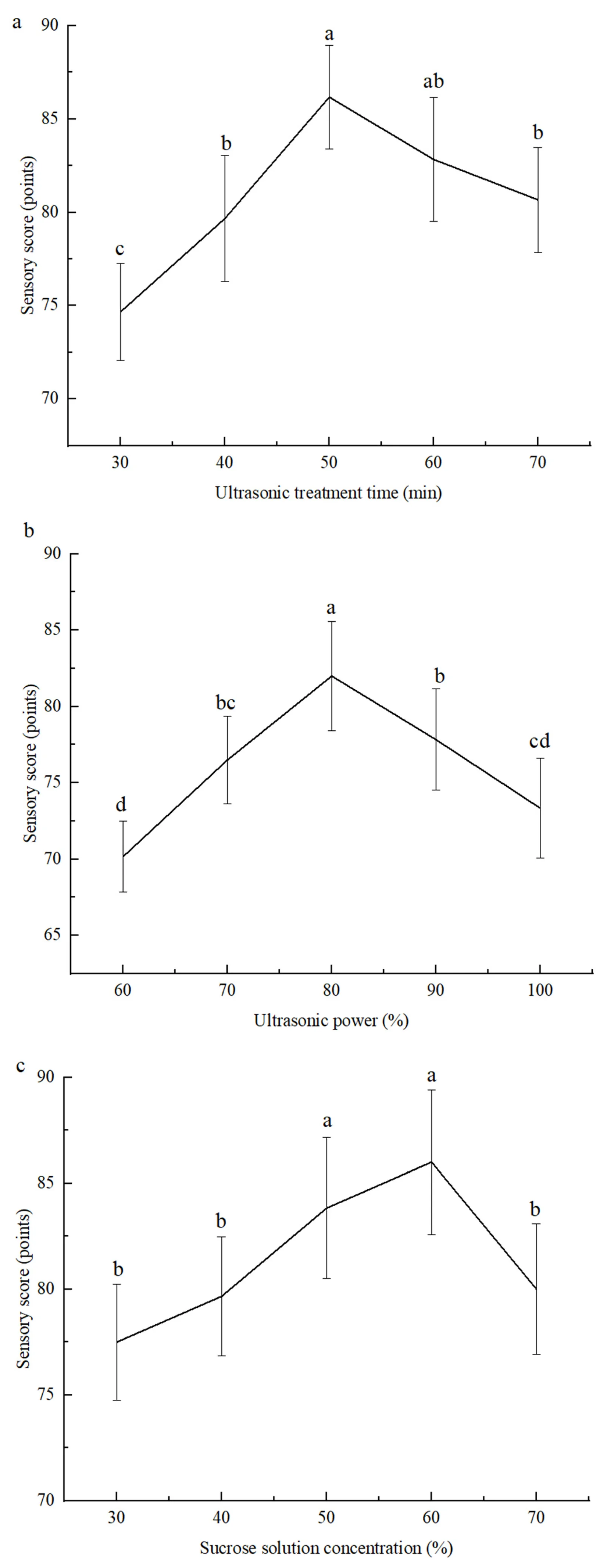
Single-factor analysis of the sensory score of candied strawberry fruit: (**a**) effects of ultrasonic treatment time; (**b**) effects of ultrasonic power; (**c**) effects of sucrose solution concentration. Note: Different lowercase letters indicate significant differences between treatments (*p* < 0.05).

The single-factor results are presented in Figure 6. The maximum sensory scores were achieved at an ultrasonic time of 50 min, an ultrasonic power of 80%, and a sugar-solution concentration of 45%. Insufficient ultrasonic treatment led to inadequate sugar penetration, whereas excessive ultrasonic exposure disrupted the fruit tissue and softened the candied fruit. Low sugar-solution concentrations limited the infiltration efficiency, whereas excessively high concentrations produced a hard texture and excessive sweetness; both impaired the sensory performance. The parameter ranges for the BBD were therefore selected as follows: ultrasonic treatment time 40–60 min, ultrasonic power 70–90%, and sugar-solution concentration 40–50%.

The quadratic regression model for US-assisted infiltration is given in Equation (2):

(2) $$Y_{2}=304.33+1.97X_{4}+5.70X_{5}+5.06X_{6}+5.00\times 10^{-4}X_{4}X_{5}-5.10\times 10^{-3}X_{4}X_{6}\;-6.00\times 10^{-3}X_{5}X_{6}-0.02{X_{4}}^{2}-0.03{X_{5}}^{2}-0.05{X_{6}}^{2}$$

where X_4_: ultrasonic treatment time (min); X_5_: ultrasonic power (%); X_6_: sugar-solution concentration (%); Y_2_: sensory score (points).

**Figure 7:**
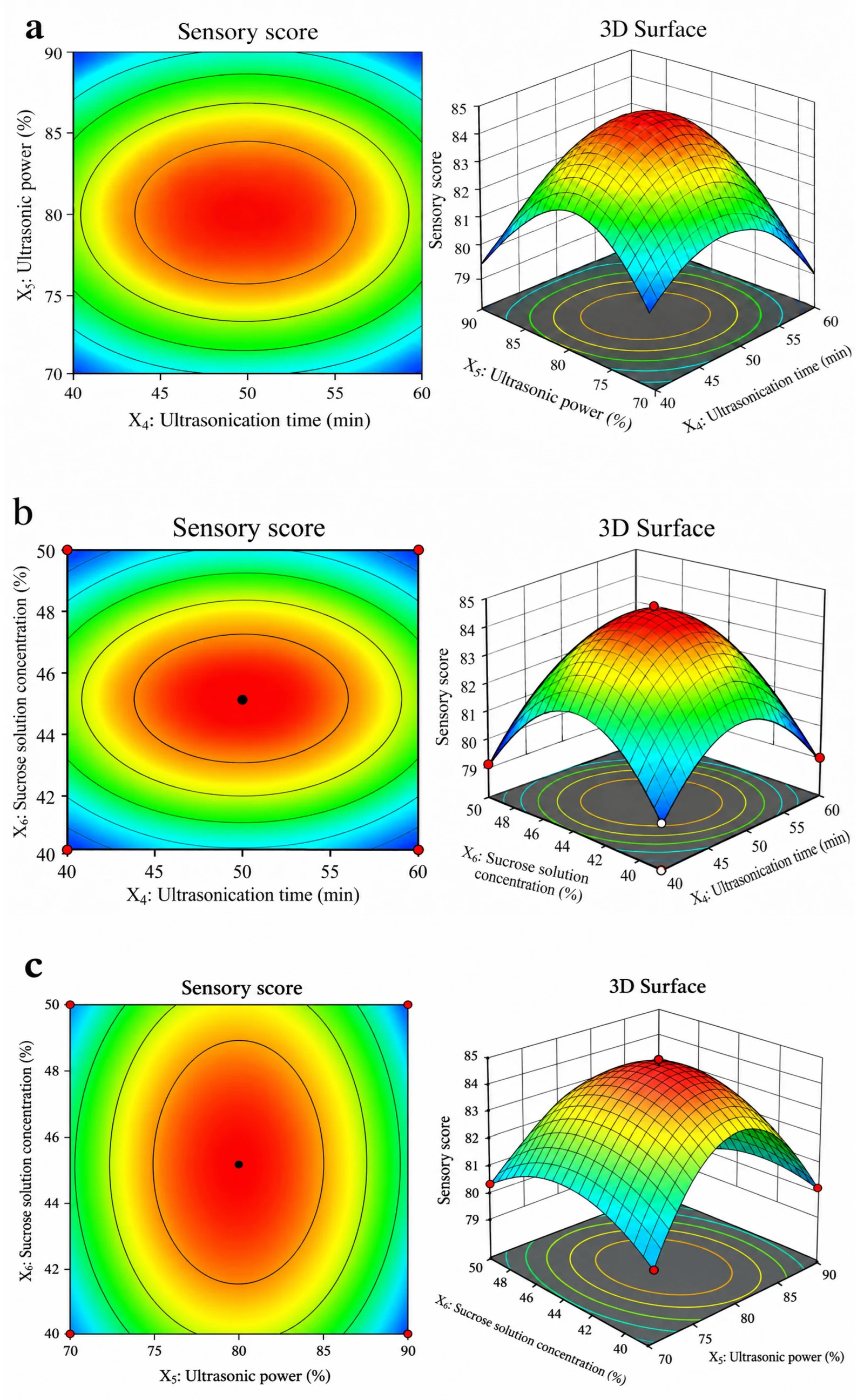
Contour plots and 3D response surface plots of sensory score for strawberry candied fruit in ultrasonic-assisted osmotic dehydration. (**a**) Ultrasonic treatment time and ultrasonic power; (**b**) ultrasonic treatment time and sucrose solution concentration; (**c**) ultrasonic power and sucrose solution concentration.

ANOVA showed that the model was extremely significant (*p* < 0.01, F = 566.37), with R^2^ = 0.99. The response surface plots are presented in Figure 7. The interaction between X_4_ and X_5_ was not significant, whereas the interactions of X_4_ with X_6_ and of X_5_ with X_6_ were highly significant. The model predicted the optimal US parameters as a treatment time of 60.00 min, an ultrasonic power of 80.00%, and a sugar-solution concentration of 45.00%, with a predicted sensory score of 84.70. The verification experiments gave measured sensory scores of 83.71, 84.25, and 84.40, with a mean of 84.12 ± 0.36 and a relative error of 0.68%.

### 3.2. BPNN Optimization Results

#### 3.2.1. BPNN for HHP-Assisted Sugar Infiltration

**Figure 8:**
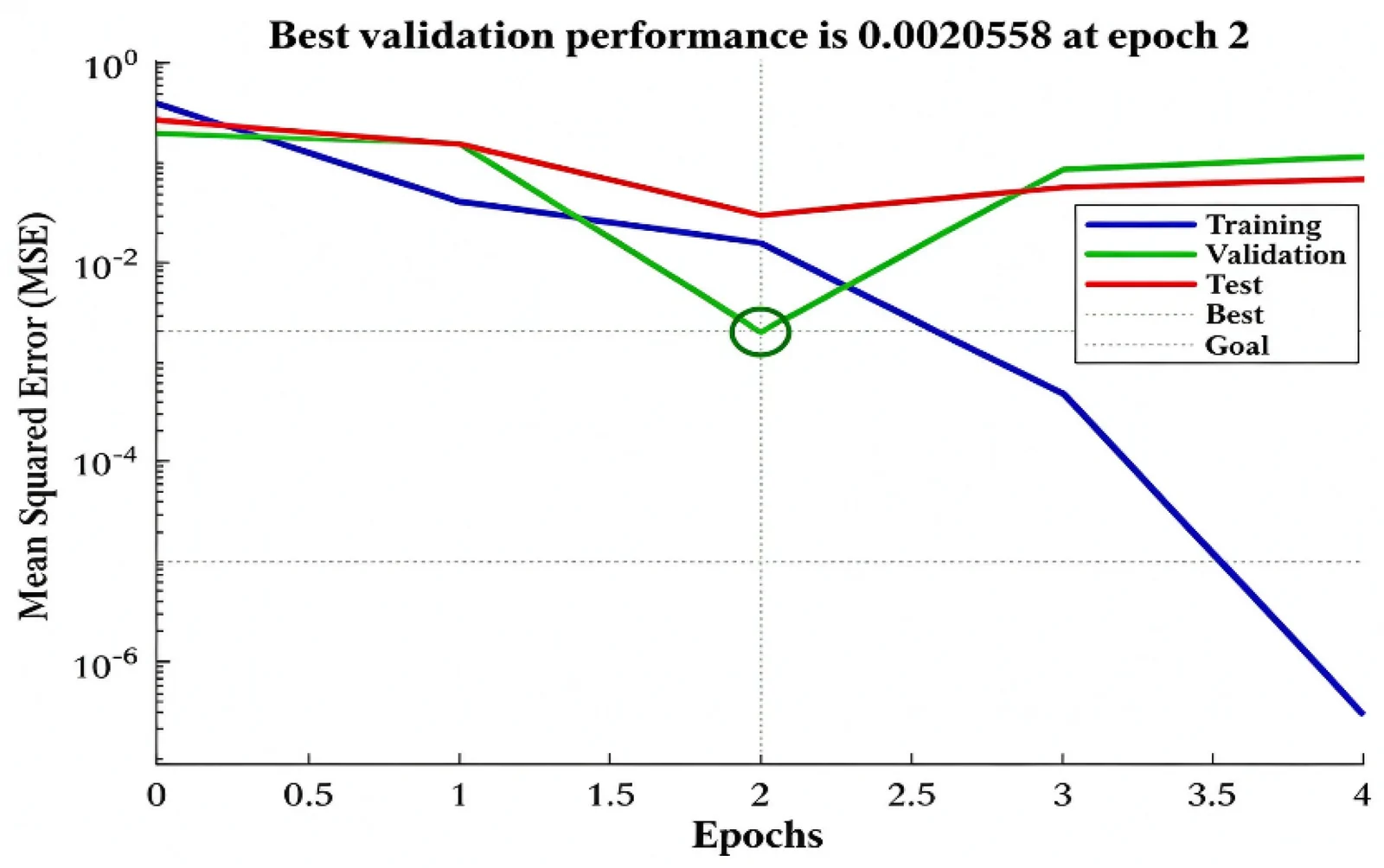
BP neural network training process for the ultra-high-pressure-assisted sugar infusion process.

**Figure 9:**
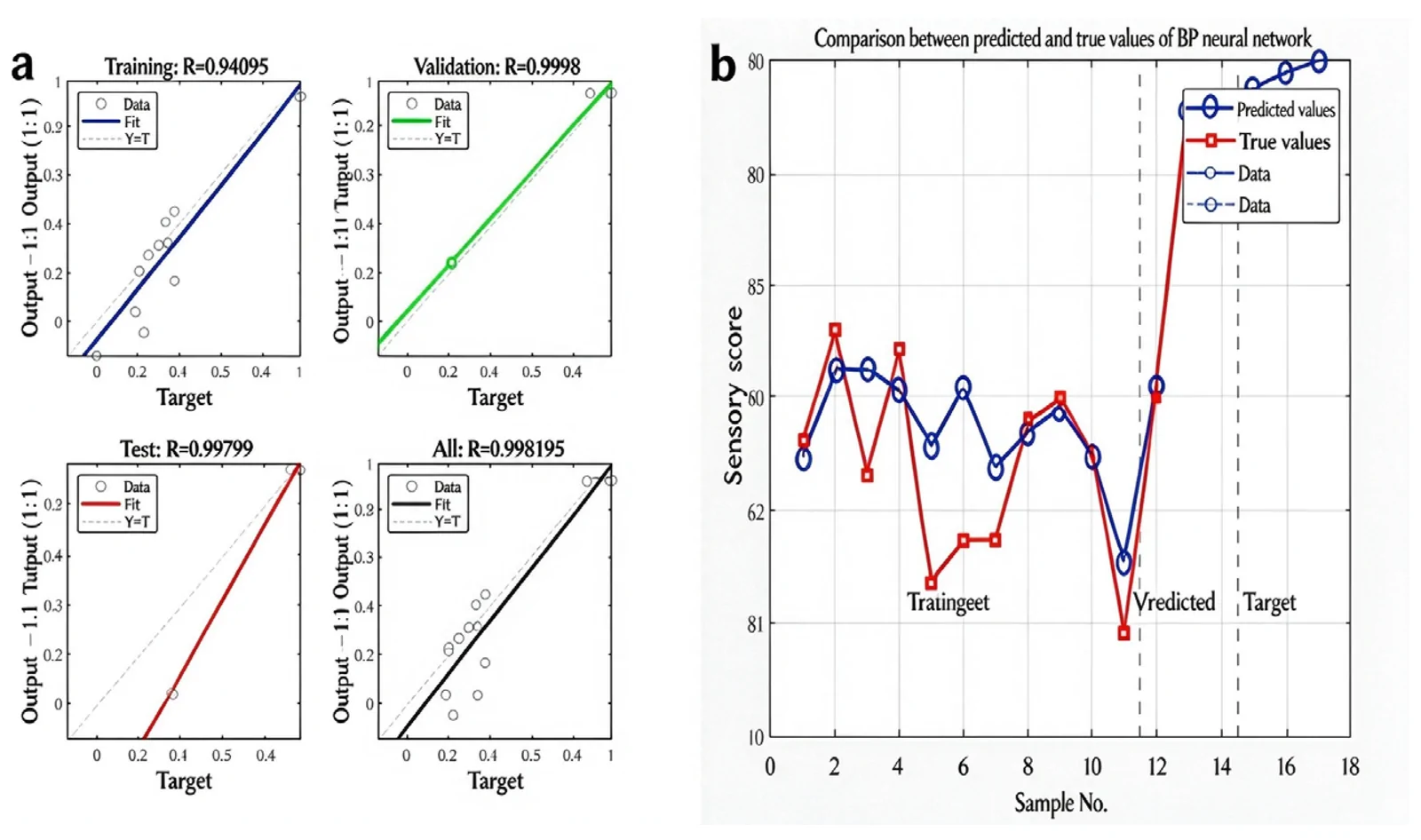
Performance of BP neural network for sensory score prediction of strawberry candied fruit under ultra-high-pressure treatment. (**a**) Correlation between predicted and target values; (**b**) Comparison of predicted and true sensory score values.

A 3-5-1 BPNN was constructed, and the optimal validation performance appeared at epoch 2 with a minimum MSE of 2.10 × 10^−3^ (Figure 8). The correlation coefficients R were 0.91 for the training set, 0.99 for the validation set, 0.99 for the test set, and 0.95 for the full dataset (Figure 9). The predicted and experimentally measured values agreed closely, which verified the stable simulation performance of the network.

The optimal HHP processing parameters predicted by the BPNN model were an infiltration time of 7.70 min, a treatment pressure of 258.00 MPa, and a sugar-solution concentration of 45.00%, with a theoretical sensory score of 86.03. The mean sensory score of the verification tests was 85.21 ± 0.39, which corresponds to a relative error of 0.95%. The comparative data for the RSM and BPNN outputs are summarized in Table 4. Although RSM showed better global fitting performance over the whole experimental domain, BPNN exhibited a lower relative error at the predicted optimal point and demonstrated a powerful nonlinear mapping and global searching capability for process optimization. The final optimal HHP-assisted sugar-infiltration parameters were therefore determined as an infiltration time of 7.70 min, a treatment pressure of 258.00 MPa, and a sugar-solution concentration of 45.00%.

#### 3.2.2. BPNN for US-Assisted Sugar Infiltration

**Figure 10:**
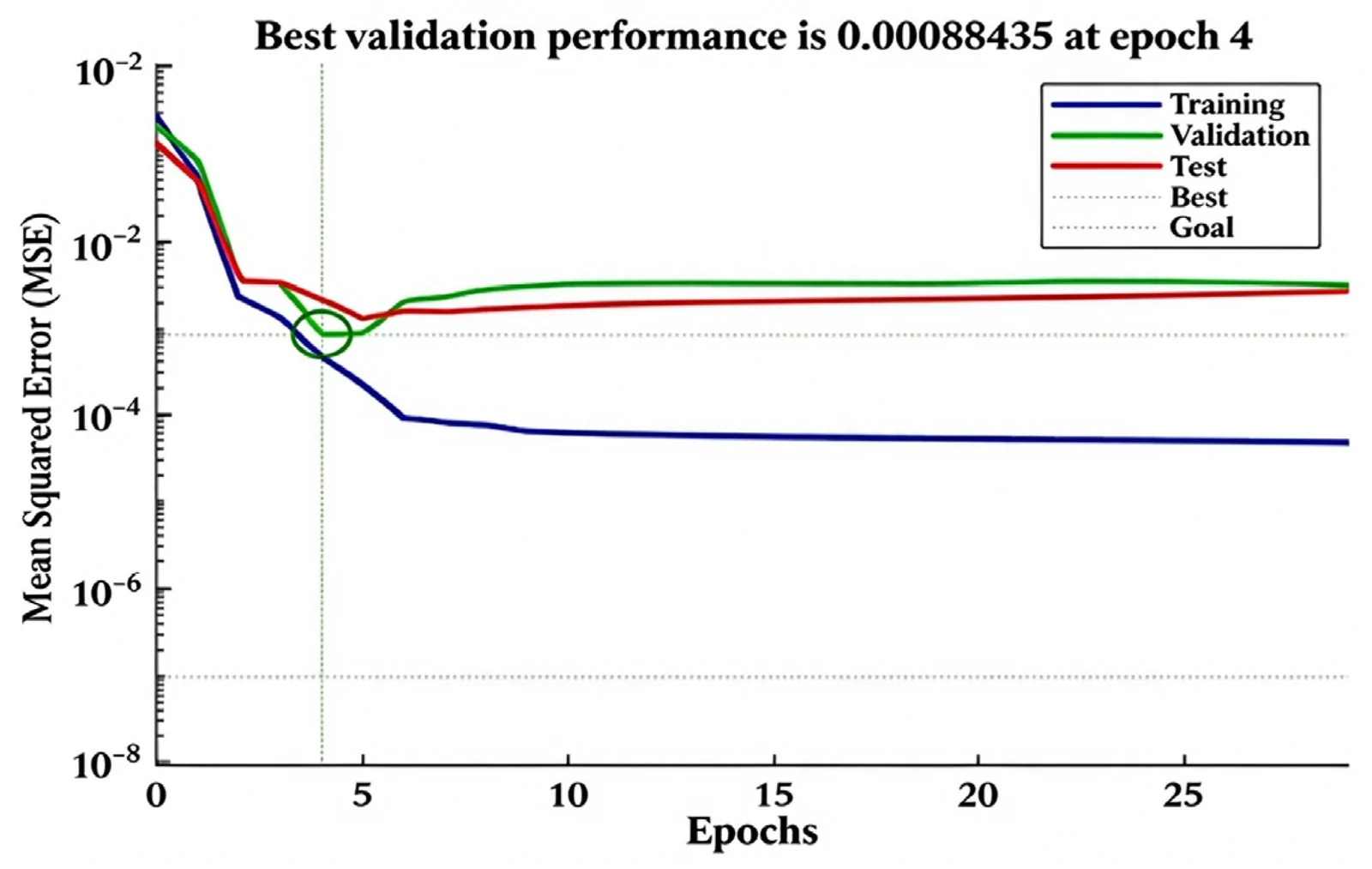
Training process of the BP neural network for the US-assisted sugar infusion process.

**Figure 11:**
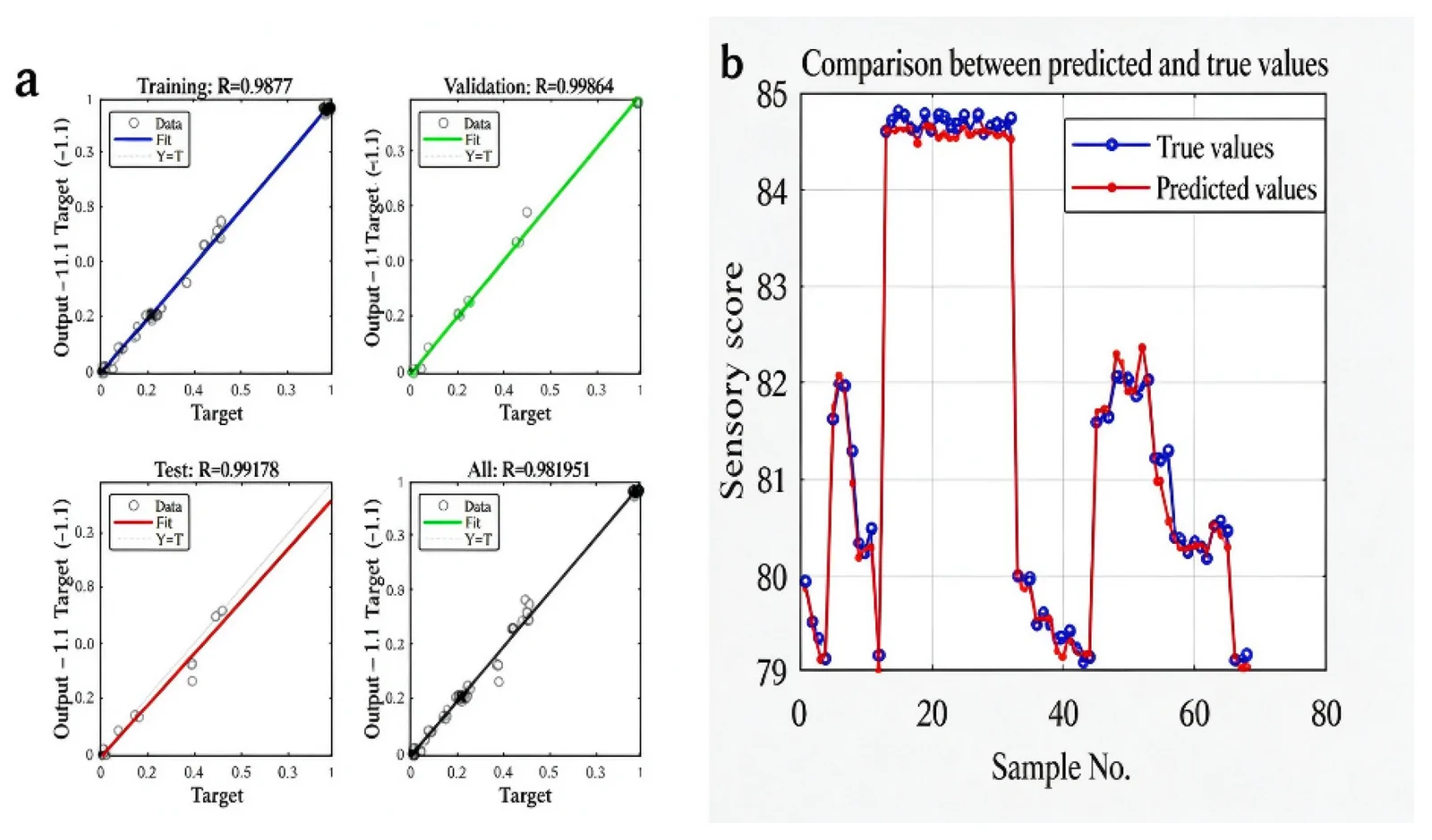
Fitting performance of BP neural network for sensory score prediction of strawberry candied fruit under ultrasonic-assisted osmotic dehydration. (**a**) Correlation between predicted and target values; (**b**) comparison of predicted and true sensory score values.

A 3-10-1 BPNN was built for US-assisted infiltration. The best validation performance occurred at epoch 4 with an MSE of 1.10 × 10^−3^ (Figure 10). The R values were 0.99 for the training set, 0.99 for the validation set, 0.97 for the test set, and 0.99 for the full dataset (Figure 11), and the predicted outputs matched the experimental data closely.

The optimal US processing parameters predicted by the BP network were a treatment time of 50.00 min, an ultrasonic power of 80.00%, and a sugar-solution concentration of 45.00%, with a theoretical sensory score of 84.71. The mean sensory score of the verification trials was 84.31 ± 0.35, which corresponds to a relative error of 0.47%. The RSM–BPNN comparison data are listed in Table 5. Although the RSM exhibited superior global fitting performance with a lower RMSE, BPNN produced a smaller relative error at the predicted optimal point and yielded a shorter ultrasonic treatment time while maintaining identical ultrasonic power and sucrose concentration. The final optimal ultrasound-assisted sugar-infiltration parameters were therefore determined as an ultrasonic time of 50.00 min, an ultrasonic power of 80.00%, and a sugar-solution concentration of 45.00%.

### 3.3. Physicochemical-Quality Comparison Across Different Processing Treatments

**Figure 12:**
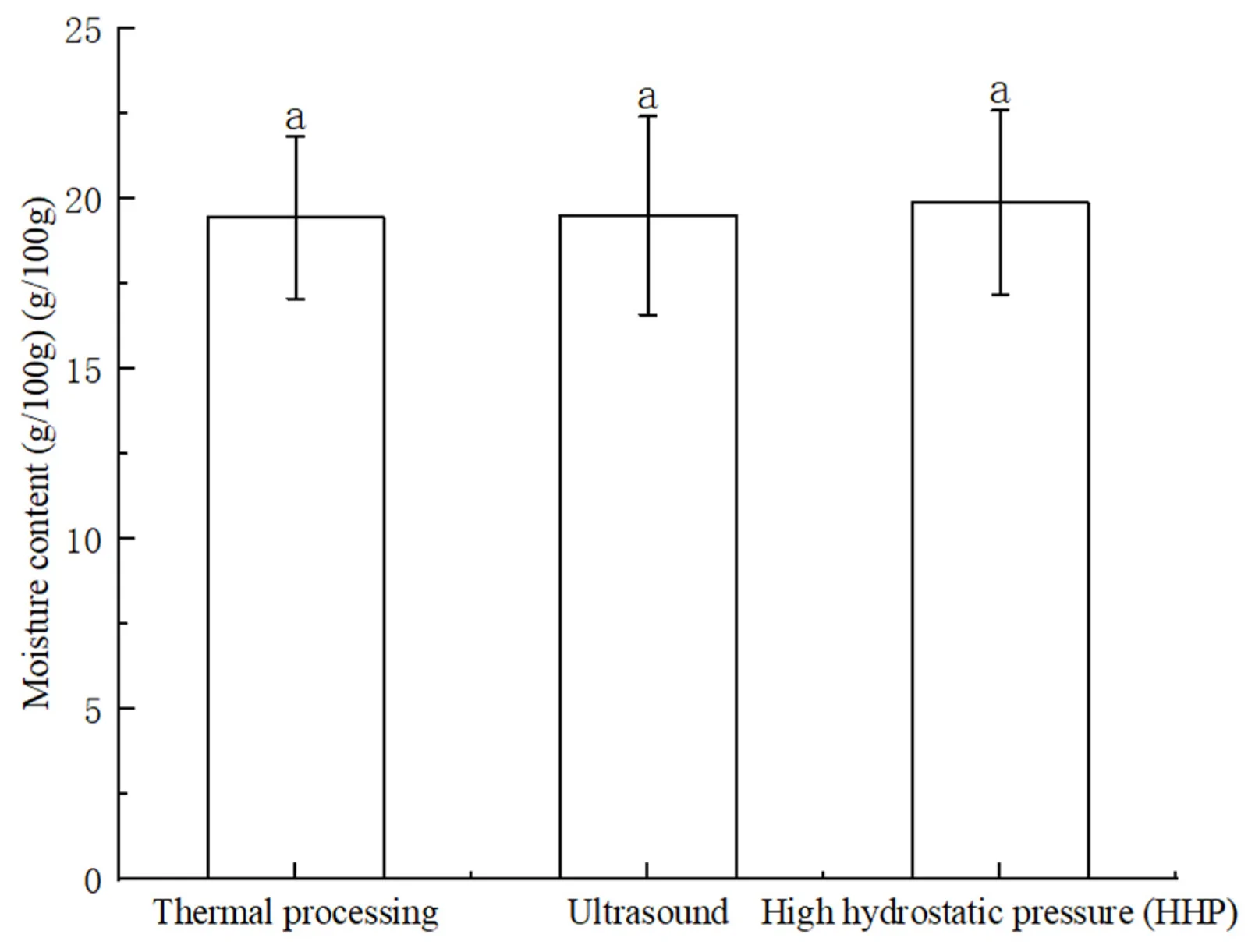
Moisture content of strawberry candied fruit under different processing methods. Note: Different lowercase letters indicate significant differences between treatments (*p* < 0.05, *n* = 3), one-way ANOVA followed by Tukey’s HSD post hoc test Values are expressed as mean ± standard deviation.

The moisture content results are presented in Figure 12. The values were 19.45 ± 0.35 g/100 g for the thermal-treatment group, 19.51 ± 0.26 g/100 g for the US-treatment group, and 19.88 ± 0.21 g/100 g for the HHP-treatment group, and no statistically significant differences were detected among the groups.

**Figure 13:**
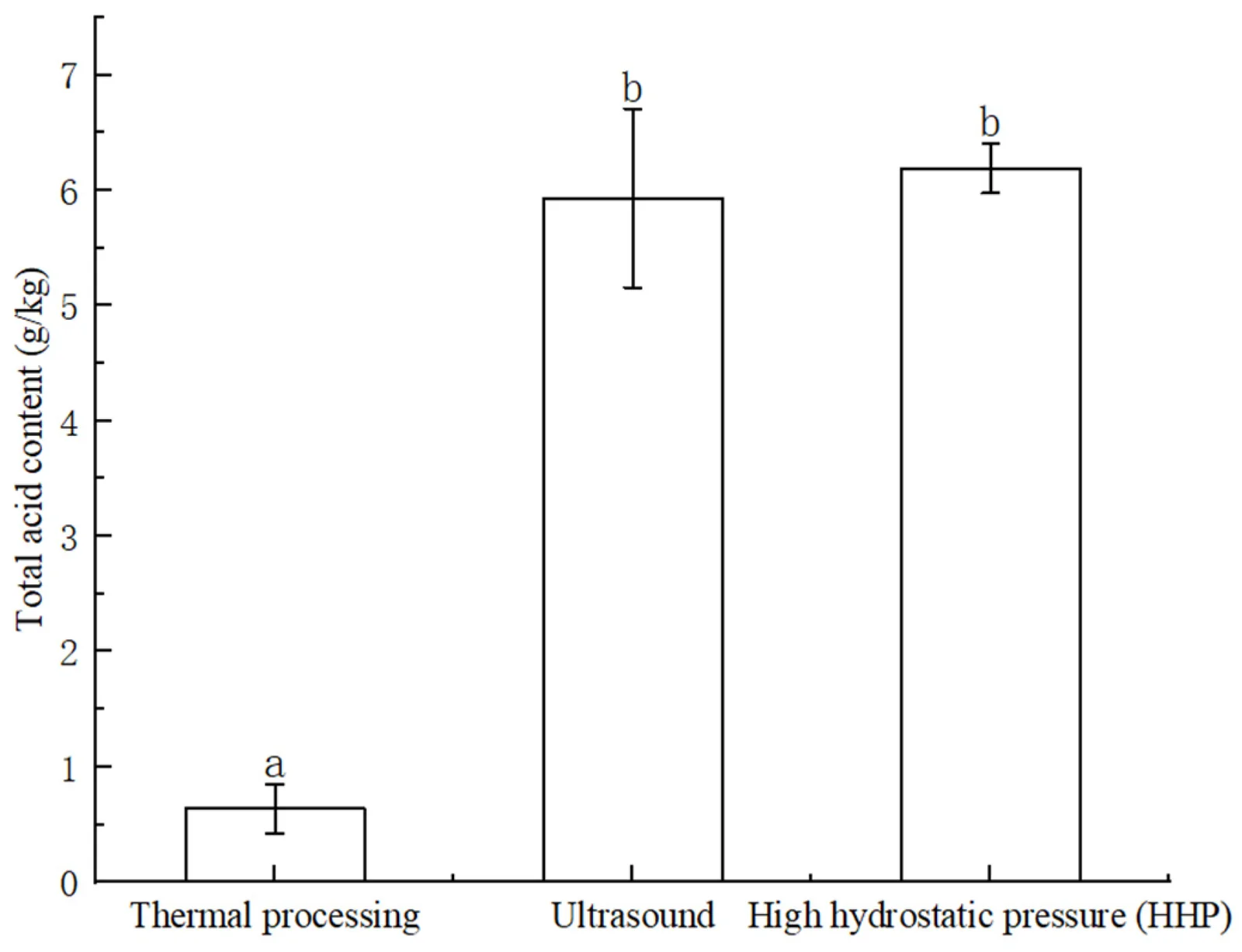
Total acid content of strawberry candied fruit under different processing methods. Note: Different lowercase letters indicate significant differences between treatments (*p* < 0.05, *n* = 3), one-way ANOVA followed by Tukey’s HSD post hoc test Values are expressed as mean ± standard deviation.

The total acid results are shown in Figure 13. Both non-thermal groups had significantly higher total acid contents than the thermal-processing group (*p* < 0.05), with the HHP-treated samples showing the highest value followed by the US-treated samples. Severe organic acid loss therefore occurred under thermal processing.

**Figure 14:**
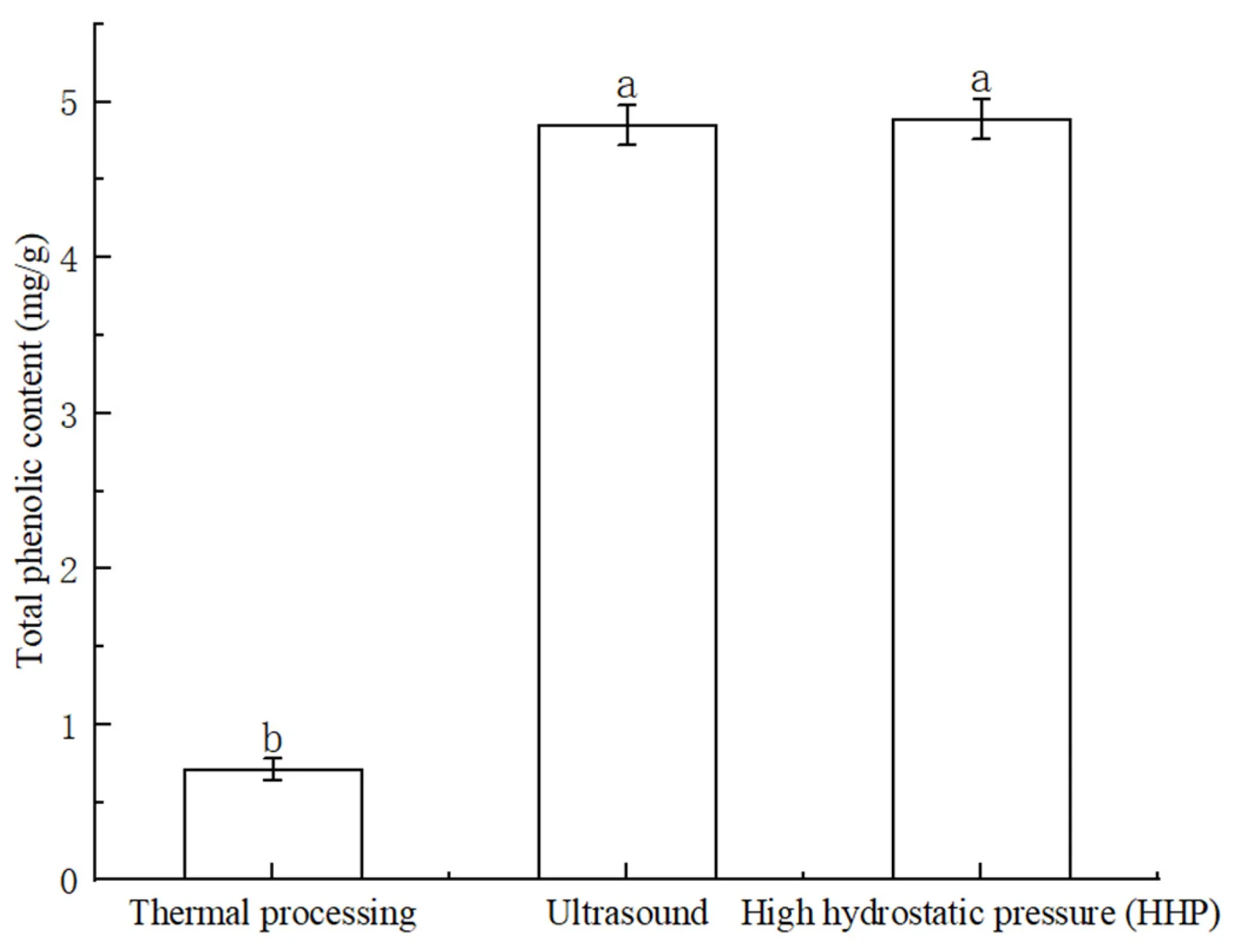
Total phenolic content of strawberry candied fruit under different processing methods. Note: Different lowercase letters indicate significant differences between treatments (*p* < 0.05, *n* = 3), one-way ANOVA followed by Tukey’s HSD post hoc test Values are expressed as mean ± standard deviation.

The total phenolic results are summarized in Figure 14. The total phenolic contents of the thermally processed samples were significantly lower than those of the US- and HHP-treated samples (*p* < 0.05), whereas no significant difference existed between the two non-thermal groups. The non-thermal treatments thus effectively protected the phenolic compounds against thermal degradation.

**Figure 15:**
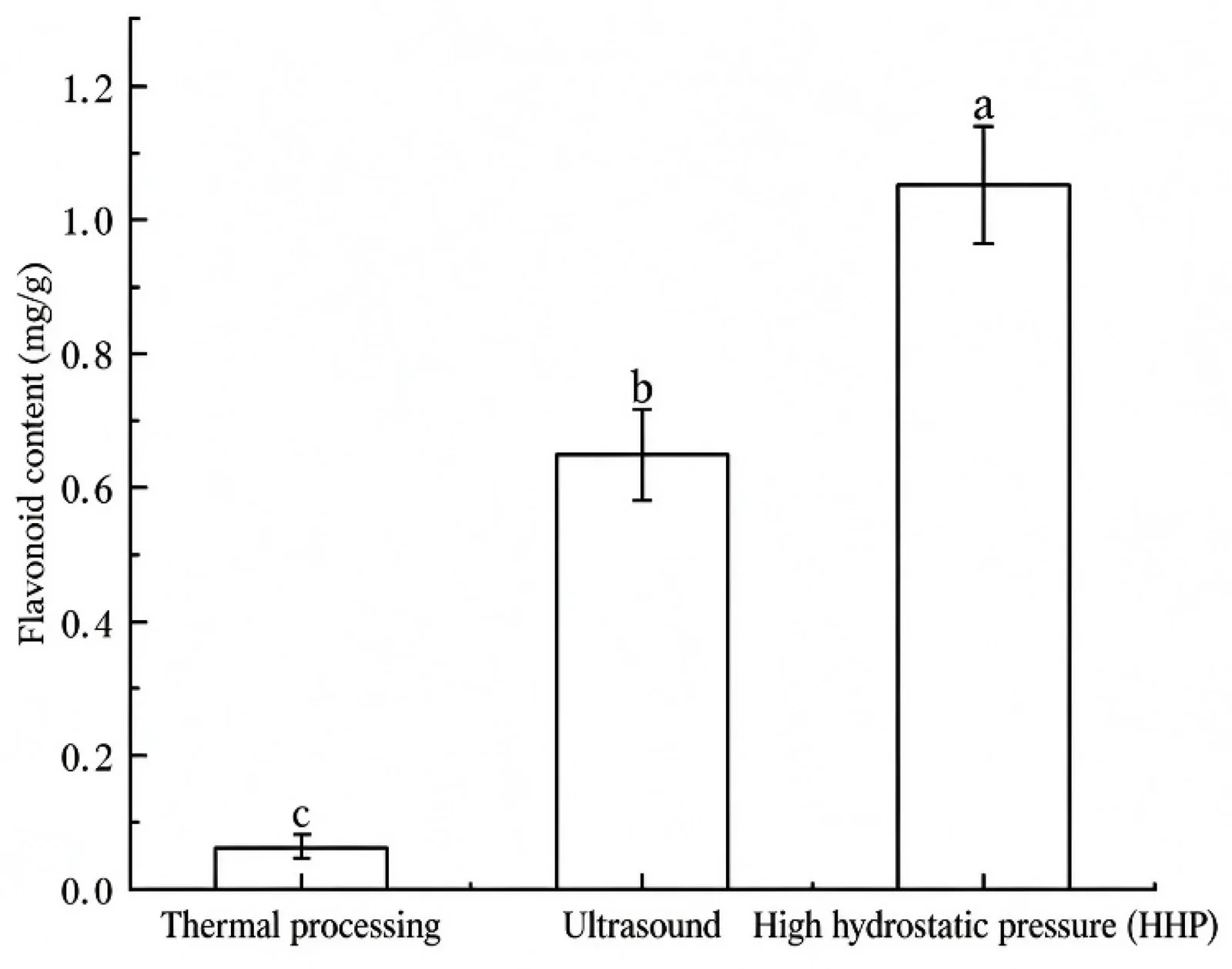
Flavonoid content of strawberry candied fruit under different processing methods. Note: Different lowercase letters indicate significant differences between treatments (*p* < 0.05, *n* = 3), one-way ANOVA followed by Tukey’s HSD post hoc test Values are expressed as mean ± standard deviation.

The total flavonoid data are given in Figure 15. The HHP-treated samples had the highest flavonoid content, followed by the US-treated samples, and the thermally processed samples had the lowest value; significant differences were observed among all three groups (*p* < 0.05). Flavonoid retention was therefore better under HHP treatment than under US treatment.

**Figure 16:**
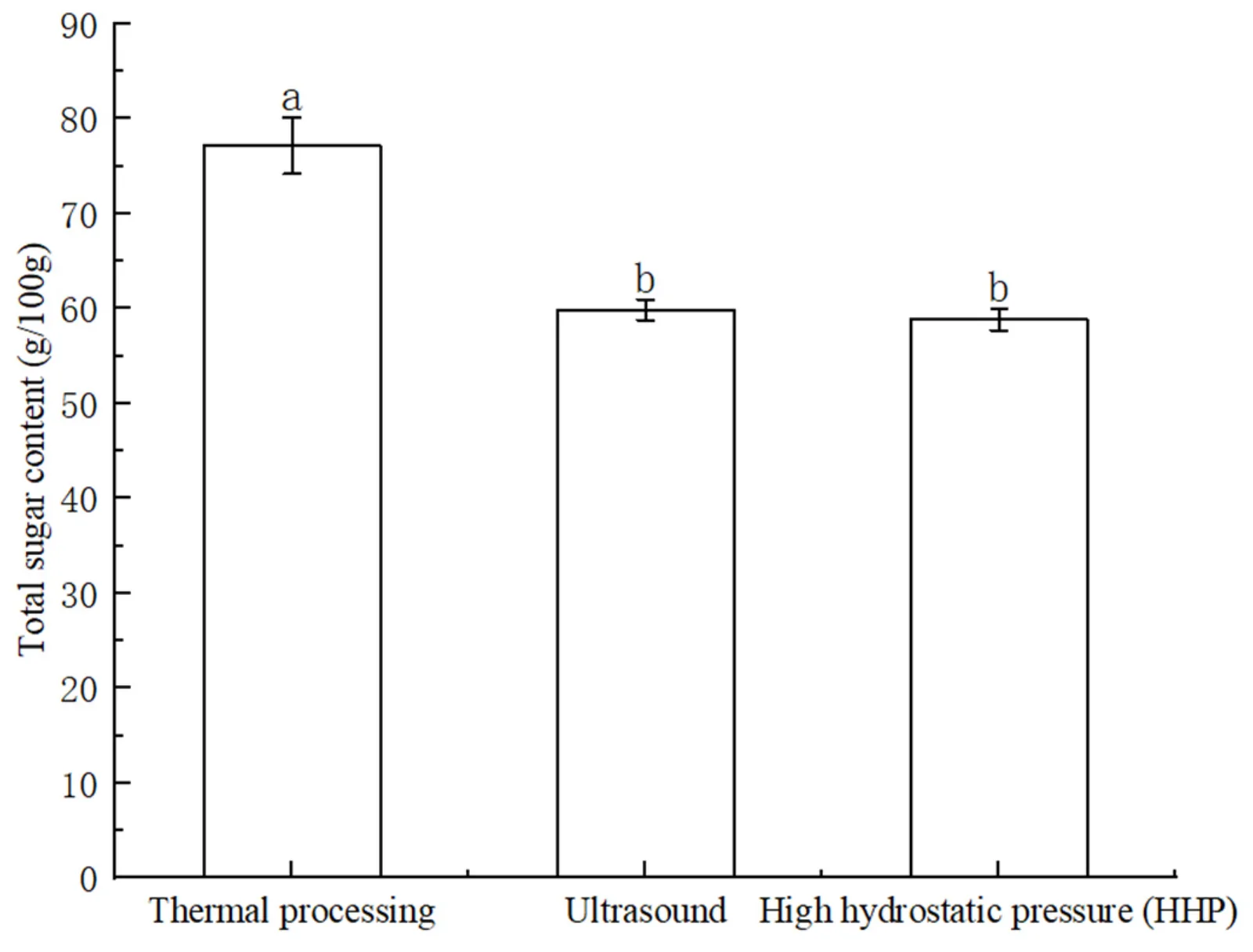
Total sugar content of strawberry candied fruit under different processing methods. Note: Different lowercase letters indicate significant differences between treatments (*p* < 0.05, *n* = 3), one-way ANOVA followed by Tukey’s HSD post hoc test Values are expressed as mean ± standard deviation.

The total sugar results are displayed in Figure 16. The US- and HHP-treated samples had significantly lower total sugar contents than the thermally processed samples (*p* < 0.05), whereas no significant difference appeared between the two non-thermal groups. All measured total sugar values complied with the national standard limit for preserved-fruit products (<85 g/100 g).

## 4. Discussion

The total phenolic contents of the HHP- and US-treated candied-strawberry samples reached 4.89 mg/g and 4.85 mg/g, respectively, which were approximately 5.8-fold higher than that of the thermally treated samples (0.71 mg/g). Similar phenolic retention under non-thermal conditions has been documented in strawberry nectar systems [21], although the magnitude of the improvement differs because of differences in the food matrix and processing workflow. This finding is consistent with previous reports on non-thermal processing [22,23], in which relatively limited phenolic enhancement was observed in most liquid-matrix studies. The more pronounced improvement obtained here originates from the specific characteristics of the present system: unlike liquid-based experiments, the candied-fruit workflow integrates sugar infiltration with drying, and the non-thermal treatment generates micro-channels within the fruit tissue [24]. These micro-channels facilitate sugar penetration and the release of bound phenolic substances, and the subsequent drying step further concentrates the phenolic compounds. In addition, high-pressure stress can activate stress-response pathways in strawberry and up-regulate the expression of key phenolic-biosynthesis-related genes, including phenylalanine ammonia-lyase and chalcone synthase [25]. Notably, the superior retention of phenolic compounds under HHP and ultrasound treatments stems from their distinct molecular mechanisms, which circumvent the dual destructive pathways induced by thermal processing. Thermal treatment triggers covalent degradation of phenolic structures and accelerates PPO- and POD-catalyzed oxidative coupling prior to enzyme inactivation, resulting in a net loss of phenolics [26]. In contrast, HHP exerts its effects mainly by disrupting non-covalent interactions within cell membranes and the tertiary conformation of proteins. It increases the permeability of the tonoplast and cell wall matrix to release vacuolar and cell-wall-bound phenolics [27]; meanwhile, an appropriate pressure level distorts the active conformation of PPO to block oxidative reactions, while preserving the carbon skeleton of phenolic molecules [28]. Ultrasound generates interconnected microchannels throughout the fruit parenchyma via acoustic cavitation, enhancing mass transfer during osmotic dehydration [24]. These microchannels promote rapid sugar infiltration, shorten the residence time of free phenolics in the aqueous tissue phase, and mitigate the leaching of phenolics into the surrounding hypertonic syrup [29,30]. Nevertheless, cavitation also produces hydroxyl radicals and transient localized hotspots that initiate phenolic oxidation [31], so the protective effect of ultrasound is only achievable within an optimized processing window. When HHP and ultrasound are applied in parallel, a synergistic protective system is established. HHP efficiently inactivates oxidative enzymes and eliminates the primary biological pathway responsible for phenolic degradation [28], whereas ultrasound improves tissue permeability to liberate bound phenolics and accelerates sugar infusion to rapidly form a hyperosmotic microenvironment that further suppresses residual enzymatic activity [24,29]. This synergy resolves the inherent limitations of individual treatments: HHP alone can release phenolics but often fails to fully suppress oxidase activity, while ultrasound alone facilitates phenolic release via cavitation at the cost of radical-mediated oxidative degradation [31]. In addition, parallel HHP-US treatment favors the retention of organic acids, maintaining a low-pH matrix that stabilizes anthocyanins in their flavylium cation form and reduces their spontaneous conversion into chalcone and other degradation products [32]. The subsequent mild drying step concentrates the recovered phenolic pool without inducing severe thermal decomposition or phenolic consumption via Maillard reactions [33]. Collectively, the final phenolic retention is governed by the combined effects of membrane permeabilization, enzyme inactivation, radical formation, solute partitioning, and pH-dependent chemical equilibria. This explains why the process windows identified by RSM and BPNN are essential for maximizing phenolic recovery and minimizing oxidative losses.

For total acid, the values of the HHP- and US-treated groups were 6.19 g/kg and 5.93 g/kg, respectively, i.e., approximately 8.7–9.7-fold higher than that of the thermal group (0.64 g/kg). Such large acid loss under thermal processing arises from the thermal degradation of organic acids and from acid consumption in Maillard-reaction pathways, which is consistent with earlier findings on the stable titratable acidity of strawberry-based matrices under HHP treatment [34]. Such variations in acid retention can be rationalized by the thermal susceptibility of the primary organic acids in strawberry. Citric and malic acids, the predominant organic acids in strawberry tissue, are heat labile and prone to decarboxylation and volatilization under prolonged heating. Meanwhile, temperature-promoted Maillard reactions further consume organic acids and trigger non-enzymatic browning [35]. Unlike thermal processing, parallel HHP–US treatment is implemented at ambient temperature for a short duration, which markedly suppresses acid losses originating from decarboxylation, volatilization, and Maillard reactions, thus enabling nearly quantitative retention of endogenous organic acids. The minor difference in acidity between the HHP and US groups is of limited practical relevance, and it may arise from the intensified solute exchange induced by acoustic cavitation during ultrasound-assisted osmotic dehydration [30]. Acid retention is highly responsive to processing conditions: elevated drying temperatures accelerate acid decarboxylation and Maillard-related consumption, extended osmotic dehydration facilitates acid leaching into the syrup [29], and a higher initial syrup pH increases the tissue pH. Any of these alterations reduces the final acid content and triggers a cascade of adverse chemical consequences, ultimately deteriorating color, flavor, and microbial safety. Therefore, titratable acid retention is not merely an individual quality attribute but a core chemical regulator governing overall product stability. The low-pH microenvironment sustained by sufficient organic acids stabilizes anthocyanins in their flavylium cation form [32], restricts the catalytic capacity of residual PPO, inhibits microbial growth, and establishes the sugar–acid balance responsible for the sweet–sour sensory perception of candied strawberry. The non-thermal groups gave total sugar values near 59 g/100 g, markedly lower than that of the thermal-processing group (77.17 g/100 g), which meets the low-sugar market demand for preserved-fruit commodities. The moisture content remained comparable across all three groups (19.4–19.9 g/100 g), which indicates that the non-thermal treatments exerted no obvious adverse effect on the fundamental physical properties of the candied-fruit products. Taken together, both HHP-assisted and US-assisted sugar infiltration delivered better comprehensive physicochemical performance than conventional thermal processing.

This work also compared the optimization performance of the RSM and BPNN models. The BPNN gave smaller relative errors than RSM in both HHP-assisted (0.95% vs. 1.15%) and ultrasound-assisted (0.47% vs. 0.68%) processing. This finding agrees with the existing literature [36,37] and confirms the inherent strengths of BPNN in nonlinear fitting and global-solution searching. The combined application of the two optimization approaches therefore improves prediction reliability and provides methodological references for the process optimization of preserved fruit.

Several limitations remain in this investigation. The microbial safety and sensory shelf-life of the products were not evaluated, and their storage stability and industrial-scale applicability require further validation. Future work should also decipher the molecular mechanisms underlying the phenolic accumulation triggered by non-thermal treatment.

## 5. Conclusions

This study applied two non-thermal auxiliary technologies, HHP and US, to the sugar infiltration of candied strawberries and further used response surface methodology and BPNN models for process optimization. The BPNN model gave smaller relative errors than the RSM model in both HHP-assisted (0.95%) and US-assisted (0.47%) processing (vs. 1.15% and 0.68%, respectively), which demonstrates its better stability and optimization capacity. The final optimal HHP-assisted sugar-infiltration parameters were an infiltration time of 7.70 min, a treatment pressure of 258.00 MPa, and a sugar-solution concentration of 45.00%, whereas the optimal US-assisted parameters were a treatment time of 50.00 min, an ultrasonic power of 80.00%, and a sugar-solution concentration of 45.00%. In terms of physicochemical quality, the non-thermally processed candied-strawberry samples exhibited significantly higher total phenolic and total flavonoid contents and a reduced total sugar content, while their moisture content remained comparable to that of the thermally processed samples. In summary, both HHP-assisted and US-assisted non-thermal sugar infiltration effectively improve the nutritional quality of candied-strawberry products, and combined with model-driven parameter optimization, they support efficient, low-consumption manufacturing, and constitute a promising technical strategy for the production of high-quality candied strawberries.

## Figures and Tables

**Table 1:** Response surface experimental factor levels for ultrahigh-pressure osmosis sugar.

Factor		Level	
	−1	0	1
X_1_ sugar infiltration time /min	4	6	8
X_2_ treatment pressure/MPa	200	250	300
X_3_ sugar-solution concentration/%	40	45	50

Note: X_1_, sugar infiltration time time X_2_, treatment pressure X_3_, sugar-solution concentration, *n* = 3, central point experiments were performed in quintuplicate.

**Table 2:** Ultrasonic sugar infiltration response surface experiment factor level table.

Factor		Level	
	−1	0	1
X_4_ Ultrasonication time/min	40	50	60
X_5_ ltrasonic power/%	70	80	90
X_6_ ucrose solution concentration/%	40	45	50

Note: X_4_, sugar infusion time; X_5_, ultrasonic power; X_6_, sucrose solution concentration. *n* = 3, central point experiments were performed in quintuplicate.

**Table 3:** Sensory evaluation of dried strawberry fruit.

Sensory Attribute	Score/Points	Grade I (15–25 Points)	Grade II (5–15 Points)	Grade III (0–5 Points)
Appearance	25	Uniform color with normal light red hue	Relatively uniform color, insufficient strawberry pigmentation	Uneven color with miscellaneous discoloration on the surface
Aroma	25	No off-odors; characteristic sweet aroma of strawberry and candied fruit	No off-odors; weak lingering sweet taste of strawberry	Off-odors present; no strawberry flavor
Sweet-sour taste	25	Well-balanced sweetness and sourness	Slightly too sour or too sweet	Excessively sour or excessively sweet
Texture	25	Moderate firmness, pleasant mouthfeel and compact texture	Acceptable firmness, relatively good mouthfeel and moderate texture	Overly high or low firmness, poor mouthfeel and loose texture

Note: Grade I, excellent quality; Grade II, acceptable quality; Grade III, unacceptable quality. Sensory evaluation was performed by a trained panel consisting of 10 assessors.

**Table 4:** Comparison of predicted and measured values optimized by response surface methodology (RSM) and back-propagation (BP) neural network model under ultra-high pressure treatment

Model	Optimal Conditions			Sensory Score/Points				
	Sugar Infusion Time/Min	Sugar Infusion Pressure/MPa	Sucrose Solution Concentration/%	Predicted Value	Measured Value	RE/%	R^2^	RMSE
RSM	6.00	250.00	45.00	85.82	84.83 ± 0.51	1.15	0.99	0.19
BPNN	7.70	258.00	45.00	86.03	85.21 ± 0.39	0.95	0.91	0.57

**Table 5:** Comparison of predicted and measured values optimized by response surface methodology (RSM) and back-propagation (BP) neural network model under ultrasonic-assisted treatment

Model	Optimal Conditions			Sensory Score/Points				
	Ultrasonic Time/min	Ultrasonic Power/%	Sugar Solution Concentration/%	Predicted Value	Measured Value	RE/%	R^2^	RMSE
RSM	60.00	80.00	45.00	84.70	84.12 ± 0.36	0.68	0.99	0.12
BPNN	50.00	80.00	45.00	84.71	84.31 ± 0.35	0.47	0.99	0.16

## Data Availability

The original contributions presented in the study are included in the article. Further inquiries can be directed to the corresponding author.

## References

[1] Newerli-Guz J., Śmiechowska M., Drzewiecka A., Tylingo R. (2023). Bioactive Ingredients with Health-Promoting Properties of Strawberry Fruit (*Fragaria* x *ananassa* Duchesne). Molecules.

[2] Shahbazi Y. (2024). Effect of Modified Atmosphere Packaging Combined with Potato Starch Coating in Improving the Shelf-Life of Fresh Strawberries. J. Nutr. Fasting Health.

[3] Kumar S., Kumar J., Tripathi J., Gupta S., Gautam S. (2022). Secondary product from strawberry (*Fragaria ananassa*) fruit for extended preservation and value addition. J. Food Sci. Technol..

[4] Demir N., Alpaslan M. (2024). Determination of impregnation parameters and volatile components in vacuum impregnated apricots. Heliyon.

[5] Pobiega K., Matys A., Trusinska M., Rybak K., Witrowa-Rajchert D., Nowacka M. (2023). The Effect of Ultrasound and Pulsed Electric Field on the Osmotic Dehydration Process of Strawberries. Appl. Sci..

[6] Nuñez-Mancilla Y., Pérez-Won M., Uribe E., Vega-Gálvez A., Di Scala K. (2013). Osmotic dehydration under high hydrostatic pressure: Effects on antioxidant activity, total phenolics compounds, vitamin C and colour of strawberry (*Fragaria vesca*). LWT—Food Sci. Technol..

[7] Shi F., Liu J., Sun A., Wang Y., Wang B. (2026). Surface Modification and Sugar Infusion of Freeze-Dried Hawthorn via Combined Vacuum- Ultrasound Treatment. Foods.

[8] Salehi F., Cheraghi R., Rasouli M. (2022). Mass transfer kinetics (soluble solids gain and water loss) of ultrasound-assisted osmotic dehydration of apple slices. Sci. Rep..

[9] Singh A., Mehta A., Singh A.P., Prabhakar P.K. (2022). Ultrasonic modulated osmotic dehydration of Aonla (*Phyllanthus emblica* L.) slices: An integrated modeling through ANN, GPR, and RSM. J. Food Process. Preserv..

[10] Zhang J., Zheng X., Xiao H., Shan C., Li Y., Yang T. (2024). Quality and Process Optimization of Infrared Combined Hot Air Drying of Yam Slices Based on BP Neural Network and Gray Wolf Algorithm. Foods.

[11] Yang T., Lai H., Cao Z., Niu Y., Xiang J., Zhang C., Shang L. (2022). Comparison of an Artificial Neural Network and a Response Surface Model during the Extraction of Selenium-Containing Protein from Selenium-Enriched *Brassica napus* L.. Foods.

[12] Ladika G., Tsiaka T., Stavropoulou N.A., Strati I.F., Sinanoglou V.J. (2024). Enhancing the nutritional value and preservation quality of strawberries through an optimized osmotic dehydration process. Appl. Sci..

[13] Ardhani I.C., Putri R.M., Falah M.A.F., Widodo K.H. (2021). Determination of production factors of dehydrated strawberries by using Taguchi method approach. IOP Conf. Ser. Earth Environ. Sci..

[14] Yang X., Xiong B., Yuan Z., Liao H., Liu X., Wu Y., Zhang S., Xiang Q. (2022). Polygalaxanthone III, an Active Ingredient in Polygala japonica Houtt., Repaired Malassezia-Stimulated Skin Injury via STAT3 Phosphorylated Activation. Molecules.

[15] Shahedi Y., Zandi M., Bimakr M. (2024). A computer vision system and machine learning algorithms for prediction of physicochemical changes and classification of coated sweet cherry. Heliyon.

[16] Zhu Y., Wang G., Tang M., Zhang J. (2022). Advances in Aging and Anti-Aging Research on Rubber-Modified Asphalt and Asphalt Mixtures. World J. Eng. Technol..

[17] Ali M.T., Mehraj S., Mir M.S., Shah I.A., Shah Z.A., El-Serehy H.A., Dar E.A., Bhat A.H., Wani S.A., Fayaz U. (2023). Deciphering the response of thirteen apple cultivars for growth, fruit morphology and fruit physico-chemical attributes during different years. Heliyon.

[18] Sharabiani V.R., Saadati N., Alizadeh F., Szymanek M. (2025). Non-destructive assessment of quality parameters in Javadi cv. Peach fruits using Vis/NIR spectroscopy and multiple regression analysis. Food Chem..

[19] Shelke G.N., Kad V.P., Yenge G.B., Kukde R.B., Kakade S.R., Al-Dalain S.Y., Haddad M.A., Abdeen A., Ibrahim S.F., Abdelkhalek A. (2023). Physicochemical attributes, antioxidant activity, and sensory responses of low-fat cheese supplemented with spray-dried Jamun juice (*Syzygium cumini* L.) powder. Front. Sustain. Food Syst..

[20] Zhang Y., Yun F., Man X., Huang D., Liao W. (2023). Effects of Hydrogen Sulfide on Sugar, Organic Acid, Carotenoid, and Polyphenol Level in Tomato Fruit. Plants.

[21] Pavón-Vargas D., Simkova K., Puzović A., Moreno-Barreto A., Gędas A., Murray H., Rabeeah I., Rincon S., Anh Truong N.Q., Zavarise A. (2025). Comparative analysis of aroma and phenolic compounds in strawberry nectar subjected to equivalent thermal, ohmic heating, high pressure, and pulsed electric fields processes at pilot scale. LWT—Food Sci. Technol..

[22] Yildiz S., Pokhrel P.R., Unluturk S., Barbosa-Cánovas G.V. (2021). Changes in Quality Characteristics of Strawberry Juice After Equivalent High Pressure, Ultrasound, and Pulsed Electric Fields Processes. Food Eng. Rev..

[23] Tsikrika K., Chu B.S., Bremner D.H., Lemos A. (2022). Effect of ultrasonic treatment on enzyme activity and bioactives of strawberry puree. Int. J. Food Sci. Technol..

[24] Xing Y., Ma Q., Wang K., Dong X., Wang S., He P., Wang J., Xu H. (2023). Non-thermal treatments of strawberry pulp: The relationship between quality attributes and microstructure. Ultrason. Sonochem..

[25] Kim T.E., Pyee J. (2025). Impact of High-Pressure Processing on Phenolic Compound Accumulation and Expression of Key Biosynthetic Genes in Harvested Strawberries. Mol. Nutr. Food Res..

[26] Olivas-Aguirre F.J., Rodrigo-García J., Martínez-Ruiz N.D.R., Cárdenas-Robles A.I., Mendoza-Díaz S.O., Álvarez-Parrilla E., González-Aguilar G.A., De la Rosa L.A., Ramos-Jiménez A., Wall-Medrano A. (2016). Cyanidin-3-O-glucoside: Physical-Chemistry, Foodomics and Health Effects. Molecules.

[27] Shahidi F., Yeo J. (2016). Insoluble-Bound Phenolics in Food. Molecules.

[28] Alifdalino S., Mohammed F., Filipa V.M.S. (2017). Strawberry puree processed by thermal,high pressure,or power ultrasound: Process energy requirements and quality modeling during storage. Food Sci. Technol. Int..

[29] Amami E., Khezami W., Mezrigui S., Badwaik L.S., Bejar A.K., Perez C.T., Kechaou N. (2017). Effect of ultrasound-assisted osmotic dehydration pretreatment on the convective drying of strawberry. Ultrason. Sonochem..

[30] Rojas M.L., Kubo M.T.K., Caetano-Silva M.E., Augusto P.E.D. (2021). Ultrasound processing of fruits and vegetables, structural modification and impact on nutrient and bioactive compounds: A review. Int. J. Food Sci. Technol..

[31] Mari A., Parisouli D.N., Krokida M. (2024). Exploring Osmotic Dehydration for Food Preservation: Methods, Modelling, and Modern Applications. Foods.

[32] Mattioli R., Francioso A., Mosca L., Silva P. (2020). Anthocyanins: A Comprehensive Review of Their Chemical Properties and Health Effects on Cardiovascular and Neurodegenerative Diseases. Molecules.

[33] Méndez-Lagunas L., Rodríguez-Ramírez J., Cruz-Gracida M., Sandoval-Torres S., Barriada-Bernal G. (2017). Convective drying kinetics of strawberry (*Fragaria ananassa*): Effects on antioxidant activity, anthocyanins and total phenolic content. Food Chem..

[34] Cao X., Zhang Y., Zhang F., Wang Y., Yi J., Liao X. (2011). Effects of high hydrostatic pressure on enzymes, phenolic compounds, anthocyanins, polymeric color and color of strawberry pulps. J. Sci. Food Agric..

[35] Lund M.N., Ray C.A. (2017). Control of Maillard Reactions in Foods: Strategies and Chemical Mechanisms. J. Agric. Food Chem..

[36] Che H., Li J., Fu H., Hu Y., Gao D., Li Z., Li Q., Xiao Y., Zhou Y., Lian M. (2026). Integration of ANN-GA optimization and multi-scale mechanistic analysis for ultrasound-assisted enzymatic extraction of flavonoids from Cortex Mori. Ultrason. Sonochem..

[37] Chamsai T., Wongwilad K., Wanyo P. (2026). Synergistic Intensification of Bioactive Compounds in Germinated Brown Rice via Ultrasonic–Enzymatic Pretreatment: Multi-Objective Modeling Using RSM, ANN–GA, and ANN–PSO. ACS Omega.

